# Novel endophytic pestalotioid species associated with *Itea* in Thailand

**DOI:** 10.3389/fcimb.2025.1532712

**Published:** 2025-04-03

**Authors:** Jutamart Monkai, Rungtiwa Phookamsak, Darbhe Jayarama Bhat, Toe Swe Zin Ei, Jianchu Xu, Saisamorn Lumyong

**Affiliations:** ^1^ Office of Research Administration, Chiang Mai University, Chiang Mai, Thailand; ^2^ Department of Biology, Faculty of Science, Chiang Mai University, Chiang Mai, Thailand; ^3^ Centre for Mountain Futures (CMF), Kunming Institute of Botany, Kunming, Yunnan, China; ^4^ Honghe Center for Mountain Futures, Kunming Institute of Botany, Chinese Academy of Sciences, Kunming, Yunnan, China; ^5^ Department of Economic Plants and Biotechnology, Yunnan Key Laboratory for Wild Plant Resources, Kunming Institute of Botany, Chinese Academy of Sciences, Kunming, Yunnan, China; ^6^ The Center for International Forestry Research and World Agroforestry (CIFOR-ICRAF) China Program, World Agroforestry (ICRAF), Kunming, Yunnan, China; ^7^ Department of Botany and Microbiology, College of Science, King Saud University, Riyadh, Saudi Arabia; ^8^ Biology Division, Vishnugupta Vishwavidyapeetam, Gokarna, India; ^9^ Center of Excellence in Microbial Diversity and Sustainable Utilization, Chiang Mai University, Chiang Mai, Thailand; ^10^ Academy of Science, The Royal Society of Thailand, Bangkok, Thailand

**Keywords:** Neopestalotiopsis, Pestalotiopsis, polyphasic taxonomic approach, Pseudopestalotiopsis, Sporocadaceae

## Abstract

Endophytic fungi are a well-known fascinating host-associated fungal group that can enhance plant growth and fitness by producing various bioactive secondary metabolites. They are an excellent source of industrial enzymes for potential secondary metabolite synthesis, which is useful in green agriculture, biotechnology, and pharmaceuticals. *Itea* is a valuable plant genus since it naturally contains rare sugar; however, endophytic fungi associated in this host have not yet been documented. In the present study, 11 strains of endophytic fungi were isolated and primarily identified as pestalotioid taxa based on morphological characteristics exhibited *in vitro*. Eleven strains of *Pestalotiopsis-*like taxa were isolated from the healthy leaves, stems, and roots of *Itea japonica* and *I. riparia* from Chiang Mai Province, Thailand. Species delimitation was based on morphology, multiloci phylogeny of a concatenated ITS, *tub2*, and *tef1-α* sequence data, and nucleotide polymorphism analyses. *Neopestalotiopsis iteae* and *Pseudopestalotiopsis iteae* are proposed as new species on *I. japonica* and *I. riparia*, respectively. *Neopestalotiopsis chrysea*, *N. haikouensis*, and *Pestalotiopsis jinchanghensis* are described as new records on *I. riparia*. Owing to the conspecific relationship based on multiloci phylogeny and identically nucleotide pairwise comparison of sufficient gene regions, several species are synonymized including *Neopestalotiopsis cercidicola* and *N. terricola* as *N. haikouensis*, *N. umbrinospora* as *N. chrysea*, and *Pestalotiopsis zhaoqingensis* as *P. jinchanghensis*. The updated phylogenetic trees, nucleotide comparisons, and morphological descriptions are herein provided and discussed for the taxonomic placements of these new species and records. This study is the first to investigate *Itea* endophytes in Thailand, and it reveals the intra- and interspecific relationships of pestalotioid species, which need to be further reevaluated because of ambiguous taxa.

## 1 Introduction

Pestalotioid fungi are generally referred to as appendage-bearing coelomycetes that have multi-septate and fusiform conidia in the family *Sporocadaceae* (*Amphisphaeriales*, Sordariomycetes), including genera *Neopestalotiopsis*, *Pestalotiopsis*, and *Pseudopestalotiopsis* (Maharachchikumbura et al., 2011; 2014; Peng et al., 2022; Razaghi et al., 2024)*. Pestalotiopsis* and allied genera are taxonomically, chemically, and ecologically diverse, which have been associated with a broad range of host plants through their life cycle as endophytes, pathogens, and saprobes (Peng et al., 2022; Giri and Pradhan, 2023; Hsu et al., 2024). The natural classification of this group based on the conidial morphology suggests concolorous median cells in *Pestalotiopsis* and *Pseudopestalotiopsis*, and versicolorous median cells in *Neopestalotiopsis* (Maharachchikumbura et al., 2014; Peng et al., 2022; Sun et al., 2023). Compared with *Pestalotiopsis*, *Pseudopestalotiopsis* has darker colored median cells (Maharachchikumbura et al., 2014; Nozawa et al., 2018; Sun et al., 2023). However, the characteristics of conidia (e.g., color, size, and shape) and conidial appendages (e.g., number, length, shape, branched or unbranched, and presence or absence of knobbed tips) are overlapping (Maharachchikumbura et al., 2014; Liu et al., 2017; Tsai et al., 2021). The multiloci phylogeny of the concatenated ITS, *tub2*, and *tef1-α* coupled with morphology has proved to be reliable for the taxonomic circumscription of pestalotioid species (Maharachchikumbura et al., 2012; 2014; Nozawa et al., 2017; 2018; Norphanphoun et al., 2019; Sun et al., 2023; Hsu et al., 2024).

Ecologically, *Pestalotiopsis-*like species are important as phytopathogens, endophytes, and saprobes and have a wide range of distribution in the temperate regions and tropics (Maharachchikumbura et al., 2012; 2014; Gu et al., 2022; Peng et al., 2022; Giri and Pradhan, 2023; Hsu et al., 2024). As plant pathogens, they cause various diseases, resulting in significant loss in various economic plants such as avocado (Fiorenza et al., 2022), eucalyptus (Diogo et al., 2021), blueberry (Zheng et al., 2023), macadamia (Prasannath et al., 2021), mangrove-associated hosts (Norphanphoun et al., 2019), pine (Monteiro et al., 2022), rose (Peng et al., 2022), and tea (Liu et al., 2017; Chen et al., 2018; Tsai et al., 2021). Endophytic pestalotioid fungi are considered as a valuable source for producing secondary metabolites with diverse chemical structures and promising antibacterial, antifungal, and anticancer properties (Xu et al., 2010; Palwe et al., 2021; Wu et al., 2022; Giri and Pradhan, 2023). Since the discovery of the antitumor drug taxol from *Pestalotiopsis microspora*, which was earlier isolated from *Taxus wallachiana* (Strobel et al., 1996), more studies have focused on *Pestalotiopsis* and allied genera.

*Itea* is a plant genus in *Iteaceae*, including approximately 21 species, widely distributed in Africa, Asia, and North America (POWO, 2024; Song and Zhang, 2024). *Itea japonica* is an edible species, native to Japan, and located in the temperate regions (Ayers et al., 2014; POWO, 2024), whereas *I. riparia* is endemic to China, Laos, Myanmar, Thailand, and Vietnam and is located in the tropical region (POWO, 2024; Song and Zhang, 2024). *Itea* species are recognized for their nutritional and medicinal value owing to the presence of rare sugars (e.g., D-allulose and allitol) and other bioactive compounds (e.g., coumarins, flavonoids, and terpenoids), which exhibited antioxidant, anti-hepatocellular carcinoma, anti-tuberculosis, and glycosidase inhibitory activities (Luo et al., 2014; Zeng et al., 2015; Lang et al., 2022; Feng et al., 2024). Previous studies have isolated some pathogenic and endophytic fungi from *Itea* species (Farr et al., 2021). However, fungi associated with *I. japonica* and *I. riparia* have not yet been reported. Therefore, it is worthy to investigate the fungi associated to this host plant as it could offer a promising resource for new discoveries of useful bioactive compounds and enzymes. As part of our study on endophytic fungi associated with *Itea* species, we recovered 11 *Pestalotiopsis-*like taxa from fresh tissues of *I. japonica* and *I. riparia* in Chiang Mai Province, Thailand. Therefore, the aims of this study are to identify these novel strains using morphological and molecular approaches and to examine their intra- and intergeneric affinities within the pestalotioid species.

## 2 Materials and methods

### 2.1 Collection and isolation of endophytic fungi

Fresh and healthy samples (viz., leaves, stems, and roots) of *Itea japonica* and *I. riparia* were collected from Chiang Mai Province, northern Thailand. The plant samples were initially rinsed with running tap water and cut into small sections (5 mm × 5 mm). The surface sterilization of plant tissues was carried out by immersing in distilled water for 1 min, 70% alcohol for 30 s, and 2% NaOCl for 30 s, in this order. Finally, the plant sections were thoroughly rinsed in sterile distilled water, air dried, and placed on potato dextrose agar (PDA) plates. After incubation at 25°C for 24–48 h, the hyphal tips grown on the margin of the plant tissues were picked up and inoculated onto new PDA plates to obtain the pure cultures. The cultures were incubated at 25°C for 1–2 months for the sporulation and morphological examination (Mattoo and Nonzom, 2022). The living cultures were deposited in the Culture Collection of Sustainable Development of Biological Resources Laboratory, Faculty of Science, Chiang Mai University, Chiang Mai Province, Thailand (SDBR-CMU) and the Kunming Institute of Botany Culture Collection, Kunming, China (KUNCC). To preserve the dried fungal samples (herbarium), cultures were dried in the hot air oven at 45–50°C for 1–2 days and deposited at the Herbarium of the Department of Biology, Faculty of Science, Chiang Mai University, Chiang Mai Province, Thailand (CMUB). The newly described taxa were registered for the Index Fungorum numbers (https://indexfungorum.org/Names/IndexFungorumRegisterName.asp; accessed on 13 November 2024).

### 2.2 Morphological observation and examination

The morphological characteristics of endophytic fungi (i.e., conidiomata, conidiophores, conidiogenous cells, and conidia) grown on PDA after 30 days were observed using a Nikon SMZ800N stereo microscope (Nikon Instruments Inc., Melville, NY, USA) and a Nikon Eclipse Ni compound microscope attached to a Nikon DS-Ri2 camera system (Nikon Instruments Inc., Melville, NY, USA). Permanent slides were prepared by adding lactoglycerol and sealed with clear nail polish. The measurements of fungal structures were carried out using a Tarosoft (R) Image Frame Work version 0.9.7 program. The photographic plates were edited and combined using Adobe Photoshop version 21.2.4 (Adobe Systems, San Jose, CA, USA).

### 2.3 DNA extraction, PCR amplification, and sequencing

The Biospin Fungus Genomic DNA Extraction Kit (BioFlux, Hangzhou, China) was used to extract DNA from 7-day-old colonies grown on PDA at 25°C. The internal transcribed spacer region of ribosomal DNA (ITS) was amplified using primers ITS5 and ITS4 (White et al., 1990); the beta-tubulin (*tub2*) was amplified using primers T1 and Bt2b (Glass and Donaldson, 1995; O’Donnell and Cigelnik, 1997); and the translation elongation factor-1 alpha (*tef1-α*) was amplified using primers EF1-728F and EF2 (O’Donnell et al., 1998; Carbone and Kohn, 1999). The PCR conditions for the three loci were set up following Sun et al. (2023) and Gao et al. (2024). For *Pseudopestalotiopsis* species, the beta-tubulin (*tub2*) was additionally amplified using primers Bt2a and Bt2b (Glass and Donaldson, 1995; O’Donnell and Cigelnik, 1997) with the PCR condition as described by Maharachchikumbura et al. (2012). The PCR amplification was performed in a 25-μL reaction volume, containing 12.5 μL of Master Mix (mixture of *EasyTaq*TM DNA Polymerase, dNTPs, and optimized buffer; Beijing TransGen Biotech Co., Ltd., Chaoyang District, Beijing, China), 8.5 μL of double-distilled water (ddH_2_O), 2 μL of DNA template, and 1 μL of each forward and reverse primer (10 μM). The purification of PCR products and DNA sequencing were conducted by TsingKe Company (Kunming City, Yunnan Province, China).

### 2.4 Phylogenetic analyses

The sequences were checked and assembled using BioEdit version 7.0.5.3 (Hall, 1999). The homogeneous sequences were obtained from relevant publications (Li et al., 2022; Zhang et al., 2022; 2024; Sun et al., 2023; Dong et al., 2023; Hsu et al., 2024) and the results of BLAST searches that were downloaded from GenBank (www.ncbi.nlm.nih.gov/blast/; accessed on 1 September 2024) (**Tables 1**–**3**). Alignments for each locus were carried out using the MAFFT v7.307 online version (Katoh et al., 2019; https://mafft.cbrc.jp/alignment/server/, accessed on 1 September 2024) and manually verified in BioEdit version 7.0.5.3 (Hall, 1999). To construct the phylogenetic analyses, maximum likelihood (ML) was previously applied for single locus, and further combined datasets of ITS, *tub2*, and *tef1-α* gene loci were analyzed by Bayesian inference (BI) and ML when the tree topology of each locus is congruent. ML analysis was generated with 1,000 bootstrap (BS) iterations and the GTRGAMMA model of nucleotide evolution using RAxML-HPC2 on XSEDE (v.8.2.12) (Stamatakis, 2014) via the online web platform CIPRES Science Gateway V3.3 (Miller et al., 2010). The best-fit substitution models were evaluated using MrModeltest v.2.3 (Nylander, 2004). BI analysis was implemented with MrBayes on XSEDE v.3.2.7a (Huelsenbeck and Ronquist, 2001; Zhaxybayeva and Gogarten, 2002; Ronquist et al., 2012) via the same platform (Miller et al., 2010). Six simultaneous Markov chains were run for 10 million generations but were automatically terminated when the standard deviation of split frequency approached 0.01. The trees were sampled every 100th generation, and the first 25% of sampled trees were removed during the burn-in phase of analyses, which were evaluated by Tracer v. 1.7 (Rambaut et al., 2018). The phylograms were displayed and modified with the FigTree v1.4.0 program (Rambaut and Drummond, 2012) and Adobe Illustrator version 24.3 (Adobe Systems, San Jose, CA, the USA). The newly generated sequences were submitted to the NCBI database to obtain GenBank accession numbers.

**Table 1:** GenBank accession numbers of *Neopestalotiopsis* strains used in phylogenetic analysis.

Taxa	Strains	GenBank accession number		
		ITS	*tub2*	*tef1-α*
*Neopestalotiopsis acrostichi*	MFLUCC 17-1754*	MK764272	MK764338	MK764316
*Neopestalotiopsis acrostichi*	MFLUCC 17-1755	MK764273	MK764339	MK764317
*Neopestalotiopsis alpapicalis*	MFLUCC 17-2544*	MK357772	MK463545	MK463547
*Neopestalotiopsis amomi*	HKAS 124563*	OP498012	OP752133	OP653489
*Neopestalotiopsis amomi*	HKAS 124564	OP498013	OP765913	OP753382
*Neopestalotiopsis aotearoa*	CBS 367.54*	NR163673	KM199454	KM199526
*Neopestalotiopsis arecacearum*	COAD 2017*	MH463406	MH460830	MH460838
*Neopestalotiopsis arecacearum*	COAD 2021	MH463409	MH460833	MH460841
*Neopestalotiopsis asiatica*	MFLUCC 12-0286*	JX398983	JX399018	JX399049
*Neopestalotiopsis australis*	CBS 114159*	KM199348	KM199432	KM199537
*Neopestalotiopsis brachiata*	MFLUCC 17-1555*	MK764274	MK764340	MK764318
*Neopestalotiopsis brasiliensis*	COAD 2166*	MG686469	MG692400	MG692402
*Neopestalotiopsis brasiliensis*	HGUP 191004	MZ724916	MZ724121	–
*Neopestalotiopsis camelliae-oleiferae*	CSUFTCC 81*	OK493585	OK562360	OK507955
*Neopestalotiopsis camelliae-oleiferae*	CSUFTCC 82	OK493586	OK562361	OK507956
*Neopestalotiopsis cavernicola*	KUMCC 20-0269*	MW545802	MW557596	MW550735
*Neopestalotiopsis cavernicola*	KUMCC 20-0332	MW581238	MW590328	MW590327
*Neopestalotiopsis chiangmaiensis*	MFLUCC 18-0113*	–	MH412725	MH388404
*Neopestalotiopsis chiangmaiensis*	MFLUCC 19-0048	MW248391	–	MW259070
*Neopestalotiopsis chrysea*	MFLUCC 12-0261*	JX398985	JX399020	JX399051
*Neopestalotiopsis chrysea*	MFLUCC 12-0262	JX398986	JX399021	JX399052
***Neopestalotiopsis chrysea***	**SDBR-CMU516/KUNCC 24-18917**	**PQ521226**	**PQ560699**	**PQ529177**
*Neopestalotiopsis chrysea* (=*N. umbrinospora)*	MFLUCC 12-0285	JX398984	JX399019	JX399050
*Neopestalotiopsis clavispora*	MFLUCC 12-0280	JX398978	JX399013	JX399044
*Neopestalotiopsis clavispora*	MFLUCC 12-0281*	JX398979	JX399014	JX399045
*Neopestalotiopsis cocoës*	MFLUCC 15-0152*	NR156312	–	KX789689
*Neopestalotiopsis coffeae-arabicae*	HGUP 4015	KF412647	KF412641	KF412644
*Neopestalotiopsis coffeae-arabicae*	HGUP 4019*	KF412649	KF412643	KF412646
*Neopestalotiopsis concentrica*	CFCC 55162/ROC 53*	OK560707	OM117698	OM622433
*Neopestalotiopsis concentrica*	ROC 137	OK560711	OM117702	OM622437
*Neopestalotiopsis cubana*	CBS 600.96*	KM199347	KM199438	KM199521
*Neopestalotiopsis cubana*	UMS01	OM320626	OM339539	OM339540
*Neopestalotiopsis dendrobii*	MFLUCC 14-0099	MK993570	MK975834	MK975828
*Neopestalotiopsis dendrobii*	MFLUCC 14-0106*	MK993571	MK975835	MK975829
*Neopestalotiopsis drenthii*	BRIP 72263a	MZ303786	MZ312679	MZ344171
*Neopestalotiopsis drenthii*	BRIP 72264a*	MZ303787	MZ312680	MZ344172
*Neopestalotiopsis egyptiaca*	CBS 140162*	KP943747	KP943746	KP943748
*Neopestalotiopsis elaeagni*	HGUP10002/GUCC 21002*	MW930716	MZ683391	MZ203452
*Neopestalotiopsis elaeagni*	HGUP10004/GUCC 21006	ON597079	ON595537	ON595535
*Neopestalotiopsis elaeidis*	MFLUCC 15-0735*	ON650690	–	ON734012
*Neopestalotiopsis ellipsospora*	MFLUCC 12-0283*	JX398980	JX399016	JX399047
*Neopestalotiopsis ellipsospora*	MFLUCC 12-0284	JX398981	JX399015	JX399046
*Neopestalotiopsis eucalypticola*	CBS 264.37*	NR163670	KM199431	KM199551
*Neopestalotiopsis eucalyptorum*	CBS 147684*	MW794108	MW802841	MW805397
*Neopestalotiopsis eucalyptorum*	CBS 147685	MW794098	MW802831	MW805398
*Neopestalotiopsis foedans*	CGMCC 3.9123*	JX398987	JX399022	JX399053
*Neopestalotiopsis foedans*	CGMCC 3.9178	JX398989	JX399024	JX399055
*Neopestalotiopsis foedans*	CGMCC 3.9202	JX398988	JX399023	JX399054
*Neopestalotiopsis formicarum*	CBS 115.83	KM199344	KM199444	KM199519
*Neopestalotiopsis formicarum*	CBS 362.72*	KM199358	KM199455	KM199517
*Neopestalotiopsis fragariae*	ZHKUCC 22-0113*	ON553410	ON569075	ON569076
*Neopestalotiopsis fragariae*	ZHKUCC 22-0114	ON651145	ON685198	ON685196
*Neopestalotiopsis guajavae*	FMB 0026*	MF783085	MH460871	MH460868
*Neopestalotiopsis guajavae*	FMB 0027	MF783084	MH460872	MH460869
*Neopestalotiopsis guajavicola*	FMB 0129*	MH209245	MH460873	MH460870
*Neopestalotiopsis hadrolaeliae*	VIC 47180*	MK454709	MK465120	MK465122
*Neopestalotiopsis haikouensis*	SAUCC 212271*	OK087294	OK104870	OK104877
*Neopestalotiopsis haikouensis*	SAUCC 212272	OK087295	OK104871	OK104878
***Neopestalotiopsis haikouensis***	**SDBR-CMU517/KUNCC 24-18918**	**PQ521227**	**PQ560700**	**PQ529178**
*Neopestalotiopsis haikouensis* (=*N*. *cercidicola*)	CFCC 70632	PP784737	PP842614	PP842626
*Neopestalotiopsis haikouensis* (=*N*. *cercidicola*)	CFCC 70624	PP784738	PP842615	PP842627
*Neopestalotiopsis haikouensis* (=*N*. *cercidicola*)	CFCC 70623	PP784739	PP842616	PP842628
*Neopestalotiopsis haikouensis* (= *N. terricola*)	CGMCC 3.23553	OP082294	OP235982	OP204796
*Neopestalotiopsis haikouensis* (= *N. terricola*)	UESTCC 22.0034	OP082295	OP235983	OP204797
*Neopestalotiopsis hispanica*	CBS 147686*	MW794107	MW802840	MW805399
*Neopestalotiopsis honoluluana*	CBS 111535	KM199363	KM199461	KM199546
*Neopestalotiopsis honoluluana*	CBS 114495*	KM199364	KM199457	KM199548
*Neopestalotiopsis hydeana*	MFLUCC 20-0132*	MW266069	MW251119	MW251129
*Neopestalotiopsis hydeana*	MFLUCC 20-0136	MW266066	MW251116	MW251126
*Neopestalotiopsis hypericin*	KUNCC 22-12597*	OP498010	OP765908	OP713768
*Neopestalotiopsis hypericin*	KUNCC 22-12598	OP498009	OP737883	OP737880
*Neopestalotiopsis iberica*	CBS 147688*	MW794111	MW802844	MW805402
*Neopestalotiopsis iberica*	CBS 147689	MW794114	MW802847	MW805403
*Neopestalotiopsis iranensis*	CBS 137767	KM074045	KM074056	KM074053
*Neopestalotiopsis iranensis*	CBS 137768*	KM074048	KM074057	KM074051
***Neopestalotiopsis iteae***	SDBR-CMU515/KUNCC 24-18919*	**PQ521228**	**PQ560701**	**PQ529179**
*Neopestalotiopsis javaensis*	CBS 257.31*	KM199357	KM199437	KM199543
*Neopestalotiopsis keteleeriae*	MFLUCC 13-0915*	KJ023087	KJ023088	KJ023089
*Neopestalotiopsis longiappendiculata*	MEAN 1315*	MW794112	MW802845	MW805404
*Neopestalotiopsis longiappendiculata*	MEAN 1316	MW794103	MW802836	MW805405
*Neopestalotiopsis lusitanica*	CBS 147692*	MW794110	MW802843	MW805406
*Neopestalotiopsis lusitanica*	MEAN 1318	MW794093	MW802826	MW805407
*Neopestalotiopsis macadamiae*	BRIP 63737c*	KX186604	KX186654	KX186627
*Neopestalotiopsis macadamiae*	BRIP 63748a	KX186612	KX186663	KX186636
*Neopestalotiopsis maddoxii*	BRIP 72266a*	MZ303782	MZ312675	MZ344167
*Neopestalotiopsis maddoxii*	BRIP 72272a	MZ303783	MZ312676	MZ344168
*Neopestalotiopsis maddoxii*	BRIP 72284a	MZ303785	MZ312678	MZ344170
*Neopestalotiopsis magna*	MFLUCC 12-0652*	KF582795	KF582793	KF582791
*Neopestalotiopsis mesopotamica*	CBS 299.74	KM199361	KM199435	KM199541
*Neopestalotiopsis mesopotamica*	CBS 336.86*	KM199362	KM199441	KM199555
*Neopestalotiopsis mianyangensis*	UESTCC 22.0006	OP082291	OP235979	OP204793
*Neopestalotiopsis mianyangensis*	CGMCC 3.23554 *	OP546681	OP672161	OP723490
*Neopestalotiopsis musae*	MFLUCC 15-0776*	NR156311	KX789686	KX789685
*Neopestalotiopsis musae*	MM3-2z9A	MW959799	MZ288737	MZ417508
*Neopestalotiopsis musae*	MM3-2z9C	MW959801	MZ288739	MZ417510
*Neopestalotiopsis natalensis*	CBS 138.41*	NR156288	KM199466	KM199552
*Neopestalotiopsis nebuloides*	BRIP 66617*	MK966338	MK977632	MK977633
*Neopestalotiopsis nebuloides*	BRIP 70567	OM417295	ON995131	ON624201
*Neopestalotiopsis olumideae*	BRIP 72273a*	MZ303790	MZ312683	MZ344175
*Neopestalotiopsis olumideae*	BRIP 72283a	MZ303791	MZ312684	MZ344176
*Neopestalotiopsis paeonia-suffruticosa*	CGMCC 3.23555 *	OP082292	OP235980	OP204794
*Neopestalotiopsis paeonia-suffruticosa*	UESTCC 22.0033	OP082293	OP235981	OP204795
*Neopestalotiopsis pandanicola*	MFLUCC 17-2261*	–	MH412720	MH388389
*Neopestalotiopsis pernambucana*	UFPE-URM 7148*	KJ792466	–	KU306739
*Neopestalotiopsis perukae*	FMB 0127*	MH209077	MH460876	MH523647
*Neopestalotiopsis perukae*	FMB 0130	MH208973	MH477871	MH523648
*Neopestalotiopsis petila*	MFLUCC 17-1737*	MK764275	MK764341	MK764319
*Neopestalotiopsis petila*	MFLUCC 17-1738	MK764276	MK764342	MK764320
*Neopestalotiopsis phangngaensis*	MFLUCC 18-0119*	MH388354	MH412721	MH388390
*Neopestalotiopsis photiniae*	MFLUCC 22-0129*	OP498008	OP752131	OP753368
*Neopestalotiopsis photiniae*	GUCC 21-0820	OP806524	OP896200	OP828691
*Neopestalotiopsis piceana*	CBS 394.48*	KM199368	KM199453	KM199527
*Neopestalotiopsis piceana*	SAUCC 210112	OK149224	OK206434	OK206436
*Neopestalotiopsis protearum*	CBS 114178*	JN712498	KM199463	KM199542
*Neopestalotiopsis protearum*	CBS 111506	MH553959	MH554618	MH554377
*Neopestalotiopsis psidii*	FMB 0028*	MF783082	MH477870	MH460874
*Neopestalotiopsis rhapidis*	GUCC 21501*	MW931620	MW980441	MW980442
*Neopestalotiopsis rhizophorae*	MFLUCC 17-1551*	MK764277	MK764343	MK764321
*Neopestalotiopsis rhododendri*	GUCC 21504	MW979577	MW980443	MW980444
*Neopestalotiopsis rhododendri*	GUCC 21505*	MW979576	MW980445	MW980446
*Neopestalotiopsis rhododendricola*	KUN-HKAS 123204*	OK283069	OK274147	OK274148
*Neopestalotiopsis rosae*	CBS 101057*	KM199359	KM199429	KM199523
*Neopestalotiopsis rosae*	ML1664	MT469940	MT469943	MT469946
*Neopestalotiopsis rosicola*	CFCC 51992*	KY885239	KY885245	KY885243
*Neopestalotiopsis rosicola*	CFCC 51993	KY885240	KY885246	KY885244
*Neopestalotiopsis samarangensis*	MFLUCC 12-0233*	NR120125	JQ968610	JQ968611
*Neopestalotiopsis saprophytica*	GD22-1	MK228998	MK360939	MK512492
*Neopestalotiopsis saprophytica*	MFLUCC 12-0282*	JX398982	JX399017	JX399048
*Neopestalotiopsis scalabiensis*	CAA1029*	MW969748	MW934611	MW959100
*Neopestalotiopsis sichuanensis*	CFCC 54338*	MW166231	MW218524	MW199750
*Neopestalotiopsis sichuanensis*	SM15-1C	MW166232	MW218525	MW199751
*Neopestalotiopsis siciliana*	AC46*	ON117813	ON209162	ON107273
*Neopestalotiopsis siciliana*	TAP18N016	LC427168	LC427169	LC427170
*Neopestalotiopsis sonneratae*	MFLUCC 17-1744*	MK764279	MK764345	MK764323
*Neopestalotiopsis steyaertii*	IMI 192475*	KF582796	KF582794	KF582792
*Neopestalotiopsis subepidermalis*	CFCC 55160/ROC 161*	OK560699	OM117690	OM622425
*Neopestalotiopsis subepidermalis*	CFCC 55161/ROC 169	OK560701	OM117692	OM622427
*Neopestalotiopsis suphanburiensis*	MFLUCC 22-0126*	OP497994	OP752135	OP753372
*Neopestalotiopsis surinamensis*	MFLUCC 22-0126	KM199351	KM199465	KM199518
*Neopestalotiopsis thailandica*	MFLUCC 17-1730*	MK764281	MK764347	MK764325
*Neopestalotiopsis thailandica*	MFLUCC 17-1731	MK764282	MK764348	MK764326
*Neopestalotiopsis vaccinii*	CAA1059*	MW969747	MW934610	MW959099
*Neopestalotiopsis vacciniicola*	CAA1054	MW969750	MW934613	MW959102
*Neopestalotiopsis vacciniicola*	CAA1055*	MW969751	MW934614	MW959103
*Neopestalotiopsis vheenae*	BRIP 72293a*	MZ303792	MZ312685	MZ344177
*Neopestalotiopsis vheenae*	BRIP 70210	MN114212	MN114214	MN114213
*Neopestalotiopsis vitis*	MFLUCC15-1265*	KU140694	KU140685	KU140676
*Neopestalotiopsis vitis*	MFLUCC 15-1270	KU140699	KU140690	KU140681
*Neopestalotiopsis xishuangbannaensis*	KUMCC 21-0424*	ON426865	OR025934	OR025973
*Neopestalotiopsis xishuangbannaensis*	KUMCC 21-0425	ON426866	OR025935	OR025974
*Neopestalotiopsis zakeelii*	BRIP 72271a	MZ303788	MZ312681	MZ344173
*Neopestalotiopsis zakeelii*	BRIP 72282a*	MZ303789	MZ312682	MZ344174
*Neopestalotiopsis zimbabwana*	CBS 111495*	JX556231	KM199456	KM199545
*Neopestalotiopsis zimbabwana*	MEAN 1329	MW794095	MW802828	MW805418
*Neopestalotiopsis zingiberis*	HGUP10001/GUCC 21001*	MW930715	MZ683390	MZ683389
*Neopestalotiopsis zingiberis*	HGUP10005/GUCC 21007	ON597078	ON595538	ON595536
*Pestalotiopsis diversiseta*	MFLUCC 12-0287	NR_120187	JX399040	JX399073
*Pestalotiopsis* sp*athulata*	CBS 356.86	KM199338	KM199423	KM199513

The newly generated sequences are indicated in bold and the new species are in red. The synonymized taxon is indicated in blue. The ex-type strains are represented as “*” and missing sequences are represented as “–”.

**Table 2:** GenBank accession numbers of *Pestalotiopsis* strains used in phylogenetic analysis.

Taxa	Strains	GenBank accession number		
		ITS	*tub2*	*tef1-α*
*Neopestalotiopsis cubana*	CBS 600.96*	KM199347	KM199438	KM199521
*Neopestalotiopsis protearum*	CBS 114178*	JN712498	KM199463	KM199542
*Pestalotiopsis abietis*	CFCC 53011*	MK397013	MK622280	MK622277
*Pestalotiopsis adusta*	ICMP 6088*	JX399006	JX399037	JX399070
*Pestalotiopsis* aff. *jesteri*	WPF-54	KT000164	–	–
*Pestalotiopsis* aff. *jesteri*	WPF-55-12G	KT000165	–	–
*Pestalotiopsis aggestorum*	LC6301*	KX895015	KX895348	KX895234
*Pestalotiopsis anacardiacearum*	IFRDCC 2397*	KC247154	KC247155	KC247156
*Pestalotiopsis anacardiacearum*	FY10-12	MK228990	MK360931	MK512484
*Pestalotiopsis anhuiensis*	CFCC 54791*	ON007028	ON005056	ON005045
*Pestalotiopsis appendiculata*	CGMCC 3.23550*	OP082431	OP185516	OP185509
*Pestalotiopsis arceuthobii*	CBS 433.65	MH554046	MH554722	MH554481
*Pestalotiopsis arceuthobii*	CBS 434.65*	KM199341	KM199427	KM199516
*Pestalotiopsis arengae*	CBS 331.92*	KM199340	KM199426	KM199515
*Pestalotiopsis arengae*	MFTU12	MT952584	MT957914	MT957939
*Pestalotiopsis australasiae*	CBS 114126*	KM199297	KM199409	KM199499
*Pestalotiopsis australis*	CBS 114193*	KM199332	KM199383	KM199475
*Pestalotiopsis australis*	MEAN 1096	MT374684	MT374709	MT374696
*Pestalotiopsis biciliata*	CBS 124463*	KM199308	KM199399	KM199505
*Pestalotiopsis brachiata*	LC2988*	KX894933	KX895265	KX895150
*Pestalotiopsis brachiata*	LC8188	KY464142	KY464162	KY464152
*Pestalotiopsis brassicae*	CBS 170.26*	KM199379	–	KM199558
*Pestalotiopsis camelliae*	MFLUCC 12-0277*	JX399010	JX399041	JX399074
*Pestalotiopsis camelliae*	NTUCC 18-001/BCRC FU31443	MT322016	MT321818	MT321917
*Pestalotiopsis camelliae-japonicae*	ZHKUCC 23-0826*	OR258040	OR251483	OR251480
*Pestalotiopsis camelliae-japonicae*	ZHKUCC 23-0827	OR258041	OR251484	OR251481
*Pestalotiopsis camelliae-oleiferae*	CSUFTCC 08*	OK493593	OK562368	OK507963
*Pestalotiopsis cangshanensis*	CGMCC 3.23544*	OP082426	OP185517	OP185510
*Pestalotiopsis castanopsidis*	CFCC 54430*	OK339732	OK358508	OK358493
*Pestalotiopsis castanopsidis*	CFCC 54384	OK339734	OK358510	OK358495
*Pestalotiopsis chamaeropis*	CBS 186.71*	KM199326	KM199391	KM199473
*Pestalotiopsis chamaeropis*	LC3609	KX894989	KX895320	KX895206
*Pestalotiopsis chamaeropis*	NTUPPMCC 21-054	OR125062	OR126308	OR126315
*Pestalotiopsis changjiangensis*	CFCC 54314*	OK339739	OK358515	OK358500
*Pestalotiopsis changjiangensis*	CFCC 52803	OK339741	OK358517	OK358502
*Pestalotiopsis chaoyangensis*	CFCC 55549*	OQ344763	OQ410584	OQ410582
*Pestalotiopsis chaoyangensis*	CFCC 58805	OQ344764	OQ410585	OQ410583
*Pestalotiopsis chiangmaiensis*	MFLUCC 22-0127*	OP497990	OP752137	OP753374
*Pestalotiopsis chiaroscuro*	BRIP 72970*	OK422510	OK423752	OK423753
*Pestalotiopsis chinensis*	MFLUCC 12-0273*	JX398995	–	–
*Pestalotiopsis clavata*	MFLUCC 12-0268*	NR120182	JX399025	JX399056
*Pestalotiopsis colombiensis*	CBS 118553*	KM199307	KM199421	KM199488
*Pestalotiopsis cyclobalanopsidis*	CFCC 54328*	OK339735	OK358511	OK358496
*Pestalotiopsis cyclobalanopsidis*	CFCC 55891	OK339736	OK358512	OK358497
*Pestalotiopsis daliensis*	CGMCC 3.23548*	OP082429	OP185518	OP185511
*Pestalotiopsis dianellae*	CBS 143421*	NR156664	MG386164	–
*Pestalotiopsis digitalis*	ICMP 5434*	KP781879	KP781883	–
*Pestalotiopsis dilucida*	LC3232*	KX894961	KX895293	KX895178
*Pestalotiopsis dilucida*	LC8184	KY464138	KY464158	KY464148
*Pestalotiopsis diploclisiae*	CBS 115587*	KM199320	KM199419	KM199486
*Pestalotiopsis disseminata*	CBS 118552	MH553986	MH554652	MH554410
*Pestalotiopsis diversiseta*	MFLUCC 12-0287*	NR120187	JX399040	JX399073
*Pestalotiopsis doitungensis*	MFLUCC 14-0090*	MK993573	MK975836	MK975831
*Pestalotiopsis dracaenae*	HGUP 4037*	MT596515	MT598645	MT598644
*Pestalotiopsis dracaenae*	MFLU 19-2757	MW114334	–	MW192201
*Pestalotiopsis dracaenicola*	MFLUCC 18-0913*	MN962731	MN962733	MN962732
*Pestalotiopsis dracontomelonis*	MFLUCC 10-0149*	NR168755	–	KP781880
*Pestalotiopsis dracontomelonis*	MFLUCC 22-0122	–	OP762672	OP753375
*Pestalotiopsis eleutherococci*	HMJAU 60189*	NR182556	–	–
*Pestalotiopsis eleutherococci*	HMJAU 60190	OL996127	OL898722	–
*Pestalotiopsis endophytica*	GUCC 21539	MZ477294	MZ868299	MZ868343
*Pestalotiopsis endophytica*	MFLUCC 18-0932*	NR172439	–	MW417119
*Pestalotiopsis ericacearum*	IFRDCC 2439*	KC537807	KC537821	KC537814
*Pestalotiopsis etonensis*	BRIP 66615*	MK966339	MK977634	MK977635
*Pestalotiopsis ficicola*	SAUCC230046*	OQ691974	OQ718749	OQ718691
*Pestalotiopsis ficicola*	SAUCC230042	OQ691972	OQ718747	OQ718689
*Pestalotiopsis ficicrescens*	GUCC 21556*	MZ477311	MZ868301	MZ868328
*Pestalotiopsis foliicola*	CFCC 54440*	ON007029	ON005057	ON005046
*Pestalotiopsis formosana*	NCYU 19-0353	MW114335	MW148260	MW192202
*Pestalotiopsis formosana*	NTUCC 17-009*	MH809381	MH809385	MH809389
*Pestalotiopsis formosana*	NTUCC 17-010/BCRC FU31632	MH809382	MH809386	MH809390
*Pestalotiopsis formosana*	NTUPPMCC 21-056	OR125064	OR126310	OR126317
*Pestalotiopsis furcata*	MFLUCC 12-0054*	NR120087	JQ683708	JQ683740
*Pestalotiopsis furcata*	LC6691	KX895030	KX895363	KX895248
*Pestalotiopsis fusoidea*	CGMCC 3.23545*	OP082427	OP185519	OP185512
*Pestalotiopsis gibbosa*	IFRD 411-014	KC537805	KC537819	KC537812
*Pestalotiopsis gibbosa*	NOF 3175*	LC311589	LC311590	LC311591
*Pestalotiopsis grevilleae*	CBS 114127*	KM199300	KM199407	KM199504
*Pestalotiopsis guangdongensis*	ZHKUCC 22-0016*	ON180762	ON221548	ON221520
*Pestalotiopsis guangdongensis*	ZHKUCC 22-0017	ON180763	ON221549	ON221521
*Pestalotiopsis guangxiensis*	CFCC 54308*	OK339737	OK358513	OK358498
*Pestalotiopsis guizhouensis*	CFCC 54803*	ON007035	ON005063	ON005052
*Pestalotiopsis hainanensis*	CNU060362	GQ869902	GQ869905	–
*Pestalotiopsis hainanensis*	PSHI2004Endo166*	DQ334863	DQ137861	–
*Pestalotiopsis hawaiiensis*	CBS 114491*	KM199339	KM199428	KM199514
*Pestalotiopsis hispanica*	CBS 115391*	NR161080	MH554640	MH554399
*Pestalotiopsis hispanica*	LS-1	OL441090	OL448307	OL448308
*Pestalotiopsis hispanica*	NTUPPMCC 18-162	OR125059	OR126305	OR126312
*Pestalotiopsis hollandica*	CBS 265.33*	KM199328	KM199388	KM199481
*Pestalotiopsis humicola*	CBS 336.97*	KM199317	KM199420	KM199484
*Pestalotiopsis hunanensis*	CSUFTCC 15*	OK493599	OK562374	OK507969
*Pestalotiopsis hydei*	MFLUCC 20-0135*	MW266063	MW251112	MW251113
*Pestalotiopsis iberica*	CAA 1004*	MW732248	MW759035	MW759038
*Pestalotiopsis inflexa*	MFLUCC 12-0270*	NR111789	JX399039	JX399072
*Pestalotiopsis intermedia*	MFLUCC 12-0259*	JX398993	JX399028	JX399059
*Pestalotiopsis italiana*	MFLUCC 12-0657*	KP781878	KP781882	KP781881
*Pestalotiopsis jesteri*	CBS 109350*	KM199380	KM199468	–
*Pestalotiopsis jiangxiensis*	LC4399*	KX895009	KX895341	KX895227
***Pestalotiopsis jinchanghensis***	**SDBR-CMU518/KUNCC 24-18920**	**PQ521229**	**PQ560702**	**PQ529180**
*Pestalotiopsis jinchanghensis*	LC6636*	KX895028	KX895361	KX895247
*Pestalotiopsis jinchanghensis* (*=P. zhaoqingensis*)	ZHKUCC 23-0825	OR233336	OR239062	OR239061
*Pestalotiopsis kaki*	KNU-PT-1804*	LC552953	LC552954	LC553555
*Pestalotiopsis kandelicola*	NCYUCC 19-0354	MT560723	MT563100	MT563102
*Pestalotiopsis kenyana*	CBS 442.67*	KM199302	KM199395	KM199502
*Pestalotiopsis knightiae*	CBS 114138*	KM199310	KM199408	KM199497
*Pestalotiopsis krabiensis*	MFLUCC 16-0260*	NR168199	MH412722	MH388395
*Pestalotiopsis kunmingensis*	PSHI2002Endo766*	AY373376	DQ333576	–
*Pestalotiopsis leucadendri*	CBS 121417*	MH553987	MH554654	MH554412
*Pestalotiopsis licualacola*	HGUP 4057*	KC492509	KC481683	KC481684
*Pestalotiopsis lijiangensis*	CFCC 50738*	KU860520	–	–
*Pestalotiopsis lijiangensis*	CFCC 50739	MH880834	–	–
*Pestalotiopsis linearis*	MFLUCC 12-0271*	NR120183	JX399027	JX399058
*Pestalotiopsis linguae*	ZHKUCC 22-0159 *	OP094104	OP186108	OP186110
*Pestalotiopsis linguae*	ZHKUCC 22-0160	OP094103	OP186107	OP186109
*Pestalotiopsis lithocarpi*	CFCC 55100*	OK339742	OK358518	OK358503
*Pestalotiopsis lithocarpi*	CFCC 55893	OK339743	OK358519	OK358504
*Pestalotiopsis loeiana*	MFLUCC 22-0123*	OP497988	OP713769	OP737881
*Pestalotiopsis lushanensis*	LC4344*	KX895005	KX895337	KX895223
*Pestalotiopsis macadamiae*	BRIP 63738b*	KX186588	KX186680	KX186621
*Pestalotiopsis malayana*	CBS 102220*	KM199306	KM199411	KM199482
*Pestalotiopsis manyueyuanani*	NTUPPMCC 18-165*	OR125060	OR126306	OR126313
*Pestalotiopsis manyueyuanani*	NTUPPMCC 22-012	OR125061	OR126307	OR126314
*Pestalotiopsis menhaiensis*	CGMCC 3.18250*	KU252272	KU252488	KU252401
*Pestalotiopsis monochaeta*	CBS 144.97*	NR147554	KM199386	KM199479
*Pestalotiopsis montellica*	MFLUCC 12-0279	JX399012	JX399043	JX399076
*Pestalotiopsis nanjingensis*	CSUFTCC 16*	OK493602	OK562377	OK507972
*Pestalotiopsis nanningensis*	CSUFTCC 10*	OK493596	OK562371	OK507966
*Pestalotiopsis neolitseae*	MFLU 18-2536	MW114336	MW148261	MW192203
*Pestalotiopsis neolitseae*	NTUCC 17-011*	MH809383	MH809387	MH809391
*Pestalotiopsis novae-hollandiae*	CBS 130973*	NR147557	KM199425	KM199511
*Pestalotiopsis oryzae*	CBS 353.69*	KM199299	KM199398	KM199496
*Pestalotiopsis pallidotheae*	MAFF 240993*	NR111022	LC311584	LC311585
*Pestalotiopsis pandanicola*	MFLUCC 16-0255*	MH388361	MH412723	MH388396
*Pestalotiopsis papuana*	CBS 331.96*	KM199321	KM199413	KM199491
*Pestalotiopsis papuana*	MFLU 19-2764	MW114337	MW296942	MW192204
*Pestalotiopsis parva*	CBS 265.37	KM199312	KM199404	KM199508
*Pestalotiopsis phoebes*	SAUCC230093*	OQ692028	OQ718803	OQ718745
*Pestalotiopsis phoebes*	SAUCC230092	OQ692027	OQ718802	OQ718744
*Pestalotiopsis photinicola*	GZCC 16-0028*	KY092404	KY047663	KY047662
*Pestalotiopsis pini*	MEAN 1092*	MT374680	MT374705	MT374693
*Pestalotiopsis pini*	CBS 127.80	MH553995	MH554664	MH554422
*Pestalotiopsis pinicola*	KUMCC 19-0183*	MN412636	MN417507	MN417509
*Pestalotiopsis piraubensis*	COAD 2165*	MH627381	MH643773	MH643774
*Pestalotiopsis portugallica*	CBS 393.48*	KM199335	KM199422	KM199510
*Pestalotiopsis portugallica*	NCYU 19-0352	MW114339	MW148263	MW192206
*Pestalotiopsis pyrrosiae-linguae*	ZHKUCC 23-0807*	OR199902	OR259258	OR259260
*Pestalotiopsis pyrrosiae-linguae*	ZHKUCC 23-0808	OR199903	OR259259	OR259261
*Pestalotiopsis rhizophorae*	MFLUCC 17-0416*	MK764283	MK764349	MK764327
*Pestalotiopsis rhododendri*	IFRDCC 2399*	KC537804	KC537818	KC537811
*Pestalotiopsis rhodomyrtus*	HGUP 4230*	KF412648	KF412642	KF412645
*Pestalotiopsis rosarioides*	CGMCC 3.23549*	OP082430	OP185520	OP185513
*Pestalotiopsis rosea*	MFLUCC 12-0258*	JX399005	JX399036	JX399069
*Pestalotiopsis sabal*	ZHKUCC 22-0031	ON180769	ON221555	ON221527
*Pestalotiopsis sabal*	ZHKUCC 22-0035*	ON180775	ON221561	ON221533
*Pestalotiopsis scoparia*	CBS 176.25*	KM199330	KM199393	KM199478
*Pestalotiopsis scoparia*	CBS 296.58	MH554026	MH554703	MH554461
*Pestalotiopsis sequoiae*	MFLUCC 13-0399*	KX572339	–	–
*Pestalotiopsis shaanxiensis*	CFCC 54958*	ON007026	ON005054	ON005043
*Pestalotiopsis shoreae*	MFLUCC 12-0314*	KJ503811	KJ503814	KJ503817
*Pestalotiopsis sichuanensis*	CGMCC 3.18244*	KX146689	KX146807	KX146748
*Pestalotiopsis silvicola*	CFCC 55296*	ON007032	ON005060	ON005049
*Pestalotiopsis smilacicola*	MFLUCC 22-0124	OP497989	OP762674	OP737879
*Pestalotiopsis smilacicola*	MFLUCC 22-0125*	OP497991	OP762673	OP753376
*Pestalotiopsis sonneratiae*	CFCC 57394*	ON114184	ON086816	ON086812
*Pestalotiopsis sonneratiae*	CFCC 57395	ON114185	ON086817	ON086813
*Pestalotiopsis* sp*atholobi*	SAUCC231201*	OQ692023	OQ718798	OQ718740
*Pestalotiopsis* sp*atholobi*	SAUCC231203	OQ692024	OQ718799	OQ718741
*Pestalotiopsis* sp*athulata*	CBS 356.86*	KM199338	KM199423	KM199513
*Pestalotiopsis* sp*athuliappendiculata*	CBS 144035*	MH554172	MH554845	MH554607
*Pestalotiopsis suae*	CGMCC 3.23546*	OP082428	OP185521	OP185514
*Pestalotiopsis telopeae*	CBS 114161*	KM199296	KM199403	KM199500
*Pestalotiopsis terricola*	CBS 141.69*	NR161084	MH554680	MH554438
*Pestalotiopsis thailandica*	MFLUCC 17-1616*	NR164471	MK764352	MK764330
*Pestalotiopsis trachycarpicola*	IFRDCC 2440*	NR120109	JQ845945	JQ845946
*Pestalotiopsis trachycarpicola*	MFLU 18-2524	MW114340	MW148264	MW192207
*Pestalotiopsis trachycarpicola*	NTUCC 18-004/BCRC FU31445	MT322019	MT321821	MT321920
*Pestalotiopsis trachycarpicola*	NTUPPMCC 18-160	OR125058	OR126304	OR126311
*Pestalotiopsis trachycarpicola*	NTUPPMCC 21-055	OR125063	OR126309	OR126316
*Pestalotiopsis tumida*	CFCC 55158*	OK560610	OM158174	OL814524
*Pestalotiopsis unicolor*	MFLUCC 120275	JX398998	JX399029	JX399063
*Pestalotiopsis unicolor*	MFLUCC 12-0276*	JX398999	JX399030	–
*Pestalotiopsis verruculosa*	MFLUCC 12-0274*	NR120185	–	JX399061
*Pestalotiopsis yanglingensis*	LC4553*	KX895012	KX895345	KX895231
*Pestalotiopsis yanglingensis*	NTUCC 18-005/BCRC FU31446	MT322020	MT321822	MT321921
*Pestalotiopsis yunnanensis*	HMAS 96359*	AY373375	–	–
*Pestalotiopsis yunnanensis*	PSHI2002Endo8171	AY526872	–	–

The newly generated sequences are indicated in bold. The synonymized taxon is indicated in blue. The ex-type strains are represented as “*” and missing sequences are represented as “–”.

**Table 3:** GenBank accession numbers of *Pseudopestalotiopsis* strains used in phylogenetic analysis.

Taxa	Strains	GenBank accession number		
		ITS	*tub2*	*tef1-α*
*Pestalotiopsis linearis*	MFLUCC 12-0271*	JX398992	JX399027	JX399058
*Pestalotiopsis trachycarpicola*	IFRDCC 2240*	NR_120109	JQ845945	JQ845946
*Pseudopestalotiopsis ampullacea*	LC6618*	KX895025	KX895358	KX895244
*Pseudopestalotiopsis annellata*	NTUCC 17-030*	MT322087	MT321889	MT321988
*Pseudopestalotiopsis avicenniae*	MFLUCC 17-0434*	MK764287	MK764353	MK764331
*Pseudopestalotiopsis camelliae-sinesis*	LC3490*	KX894985	KX895316	KX895202
*Pseudopestalotiopsis celtidis*	GUCC 21599*	OL423535	OL439010	OL439012
*Pseudopestalotiopsis chinensis*	LC3011*	KX894937	KX895269	KX895154
*Pseudopestalotiopsis cocos*	CBS 272.29*	KM199378	KM199467	KM199553
*Pseudopestalotiopsis curvatispora*	MFLUCC 17-1722*	MK764288	MK764354	MK764332
*Pseudopestalotiopsis dawaina*	MM14 F0015*	LC324750	LC324751	LC324752
*Pseudopestalotiopsis elaeidis*	CBS 413.62*	MH554044	MH554720	MH554479
*Pseudopestalotiopsis gilvanii*	INPA 2913*	MN385951	MN385954	MN385957
*Pseudopestalotiopsis gilvanii*	INPA 2914	MN385952	MN385955	MN385958
*Pseudopestalotiopsis ignota*	NN 42909*	KU500020	–	KU500016
*Pseudopestalotiopsis indica*	CBS 459.78*	KM199381	KM199470	KM199560
*Pseudopestalotiopsis indocalami*	GUCC 21600*	OL423536	OL439011	OL439013
***Pseudopestalotiopsis iteae***	SDBR-CMU514/KUNCC 24-18921*	**PQ521230**	**PQ560703**	**PQ529181**
***Pseudopestalotiopsis iteae***	**SDBR-CMU519/KUNCC 24-18922**	**PQ521231**	**PQ560704**	**PQ529182**
***Pseudopestalotiopsis iteae***	**SDBR-CMU520/KUNCC 24-18923**	**PQ521232**	**PQ560705**	**PQ529183**
***Pseudopestalotiopsis iteae***	**SDBR-CMU521/KUNCC 24-18924**	**PQ521233**	**PQ560706**	**PQ529184**
***Pseudopestalotiopsis iteae***	**SDBR-CMU522/KUNCC 24-18925**	**PQ521234**	**PQ560707**	**PQ529185**
***Pseudopestalotiopsis iteae***	**SDBR-CMU523/KUNCC 24-18926**	**PQ521235**	**PQ560708**	**PQ529186**
***Pseudopestalotiopsis iteae***	**SDBR-CMU524/KUNCC 24-18927**	**PQ521236**	**PQ560709**	**PQ529187**
*Pseudopestalotiopsis iteae* (as *Ps. theae*)	NTUCC 18-067	MT322086	MT321888	MT321987
*Pseudopestalotiopsis ixorae*	NTUCC 17-001.1*	MG816316	MG816326	MG816336
*Pseudopestalotiopsis kawthaungina*	MM14 F0083*	LC324753	LC324754	LC324755
*Pseudopestalotiopsis kubahensis*	UMAS-KUB-P20*	KT006749	–	–
*Pseudopestalotiopsis myanmarina*	NBRC 112264*	LC114025	LC114045	LC114065
*Pseudopestalotiopsis rhizophorae*	MFLUCC 17-1560*	MK764291	MK764357	MK764335
*Pseudopestalotiopsis simitheae*	MFLUCC 12-0121*	KJ503812	KJ503815	KJ503818
*Pseudopestalotiopsis solicola*	CBS 386.97*	NR_161086	MH554715	MH554474
*Pseudopestalotiopsis* sp.	SD012	JQ683720	JQ683704	JQ683736
*Pseudopestalotiopsis* sp.	NBRC112267	LC114030	LC114050	LC114070
*Pseudopestalotiopsis* sp.	NBRC112268	LC114031	LC114051	LC114071
*Pseudopestalotiopsis* sp.	NBRC112258	LC114036	LC114056	LC114076
*Pseudopestalotiopsis* sp.	NBRC112259	LC114039	LC114059	LC114079
*Pseudopestalotiopsis* sp.	MAFF 238515	LC114040	LC114060	LC114080
*Pseudopestalotiopsis taiwanensis*	NTUCC 17-002.1*	MG816319	MG816329	MG816339
*Pseudopestalotiopsis thailandica*	MFLUCC 17-1724*	MK764292	MK764358	MK764336
*Pseudopestalotiopsis theae*	MFLUCC 12-0055*	JQ683727	JQ683711	JQ683743
*Pseudopestalotiopsis theae*	MFLUCC 22-0128	OP497993	OP752136	OP753377
*Pseudopestalotiopsis theae*	SC011	JQ683726	JQ683710	JQ683742
*Pseudopestalotiopsis vietnamensis*	NBRC 112252*	LC114034	LC114054	LC114074

The newly generated sequences are indicated in bold and the new species are in red. The synonymized taxon is indicated in blue. The ex-type strains are represented as “*” and missing sequences are represented as “–”.

## 3 Results

### 3.1 Multiloci phylogenetic analyses

**Figure 1:**
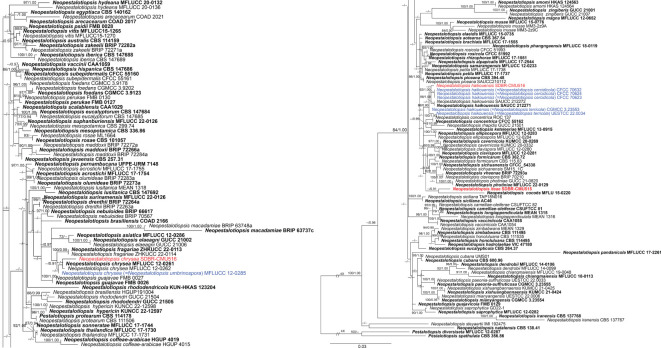
Phylogram constructed by maximum likelihood (ML) analyses of combined ITS, *tub2*, and *tef1-α* sequence dataset of *Neopestalotiopsis*. Bootstrap support valued for ML and Bayesian posterior probabilities (BYPP) equal to or greater than 70% and 0.90 are shown above the nodes as ML/BYPP. The new isolates are indicated in red and the ex-type strains are in bold. The synonymized taxon is indicated in blue. The tree is rooted with *Pestalotiopsis diversiseta* (MFLUCC 12-0287) and *P.* sp*athulata* (CBS 356.86).

Analysis 1: the combined sequence dataset of ITS, *tub2*, and *tef1-α* comprised 158 *Neopestalotiopsis* strains and two outgroup taxa, *Pestalotiopsis diversiseta* (MFLUCC 12-0287) and *P.* sp*athulata* (CBS 356.86) (**Figure 1**). The aligned dataset consisted of a total 1,896 characters with gaps (ITS: 1–541 bp, *tub2*: 542–1340 bp, *tef1-α*: 1341–1896 bp). The best-scoring RAxML tree was selected to represent the relationships among taxa, with a final likelihood value of −11,777.047879. The matrix contained 944 distinct alignment patterns, with 17.98% undetermined characters or gaps. Estimated base frequencies were as follows: A = 0.235628, C = 0.271348, G = 0.215132, and T = 0.277892; substitution rates were AC = 0.918956, AG = 2.618624, AT = 1.144576, CG = 0.760024, CT = 3.458802, and GT = 1.000000; gamma distribution shape parameter *α* = 0.361998; Tree-Length = 1.334915. For BI analysis, the best-fitting model of each locus and the final average standard deviation of split frequencies are shown in **Table 4**. Similar tree topologies were acquired by ML and Bayesian posterior probability (BYPP) analyses. The phylogenetic tree revealed that a new species, *Neopestalotiopsis iteae* (SDBR-CMU515), formed an independent branch sister to *N. cocoës* (0.91 BYPP; **Figure 1**). The new strain, SDBR-CMU517, is closely related to *N. cercidicola, N. haikouensis*, and *N. terricola* (**Figure 1**), whereas the new strain, SDBR-CMU516, grouped with *N. chrysea* and *N. umbrinospora* (**Figure 1**).

**Table 4:** The best-fit substitution model and the average standard deviation of split frequencies resulting from the Bayesian inference (BI) analysis. .

Analysis	Average standard deviation of split frequencies	Model		
		ITS	*tub2*	*tef1-α*
1. *Neopestalotiopsis*	0.009969	HKY+I+G	GTR+I+G	HKY+G
2. *Pestalotiopsis*	0.009998	GTR+I+G	HKY+I+G	GTR+I+G
3. *Pseudopestalotiopsis*	0.009889	HKY+G	HKY+G	GTR+I+G

**Figure 2:**
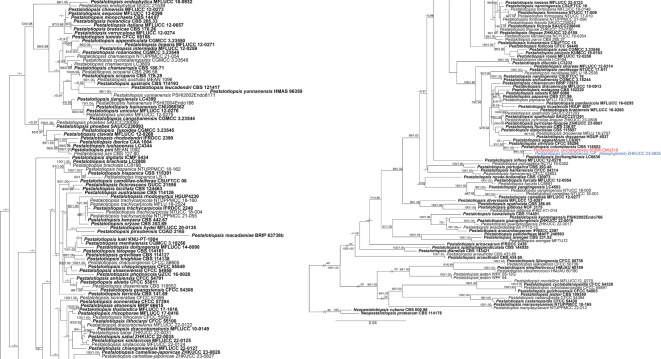
Phylogram constructed by maximum likelihood (ML) analyses of combined ITS, *tub2*, and *tef1-α* sequence dataset of *Pestalotiopsis*. Bootstrap support valued for ML and Bayesian posterior probabilities (BYPP) equal to or greater than 70% and 0.90 are shown above the nodes as ML/BYPP. The new isolates are indicated in red and the ex-type strains are in bold. The synonymized taxon is indicated in blue. The tree is rooted with *Neopestalotiopsis cubana* (CBS 114178) and *N. protearum* (ZHKUCC 23-0825).

Analysis 2: the combined sequence dataset of ITS, *tub2*, and *tef1-α* comprised 186 *Pestalotiopsis* strains and two outgroup taxa, *Neopestalotiopsis cubana* (CBS 114178) and *N. protearum* (ZHKUCC 23-0825) (**Figure 2**). The aligned dataset consisted of a total 1,959 characters with gaps (ITS: 1–604 bp, *tub2*: 605–1399 bp, *tef1-α*: 1400–1959 bp). The best-scoring RAxML tree was selected to represent the relationships among taxa, with a final likelihood value of −19,588.100995. The matrix contained 1,090 distinct alignment patterns, with 23.02% undetermined characters or gaps. Estimated base frequencies were as follows: A = 0.238520, C = 0.293691, G = 0.214954, and T = 0.252835; substitution rates were AC = 1.093468, AG = 2.897598, AT = 1.166594, CG = 1.024049, CT = 4.038506, and GT = 1.000000; gamma distribution shape parameter *α* = 0.338893, Tree-Length = 2.486656. For BI, the best-fitting model of each locus and the final average standard deviation of split frequencies are shown in **Table 4**. Similar tree topologies were acquired from ML and BYPP analyses. In the phylogenetic tree (**Figure 2**), the new strain, SDBR-CMU518, clustered together with *Pestalotiopsis jinchanghensis* and *P. zhaoqingensis* (86% ML and 1.00 BYPP; **Figure 2**).

**Figure 3:**
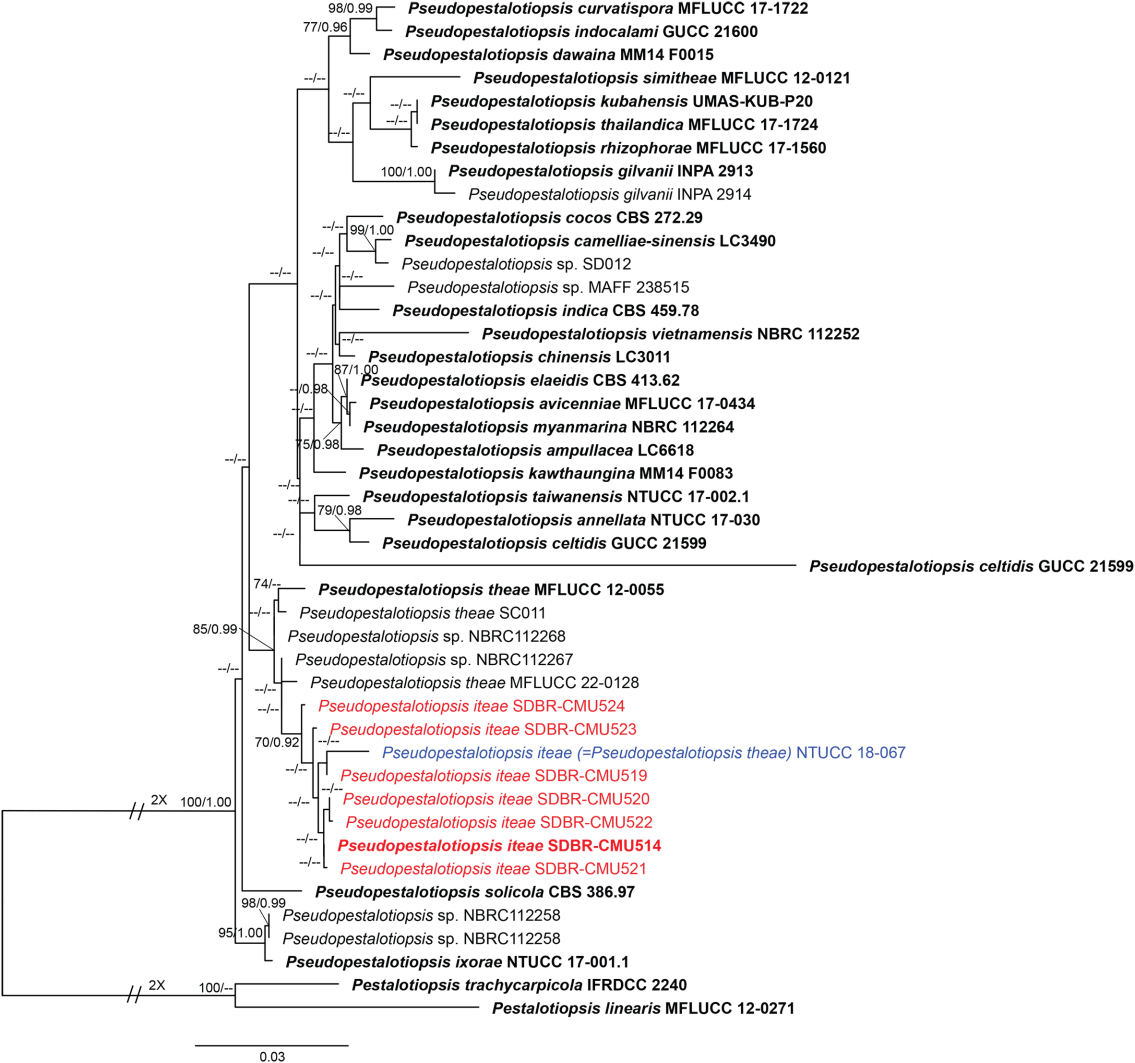
Phylogram constructed by maximum likelihood (ML) analyses of combined ITS, *tub2*, and *tef1-α* sequence dataset of *Pseudopestalotiopsis*. Bootstrap support valued for ML and Bayesian posterior probabilities (BYPP) equal to or greater than 70% and 0.90 are shown above the nodes as ML/BYPP. The new isolates are indicated in red and the ex-type strains are in bold. The synonymized taxon is indicated in blue. The tree is rooted with *Pestalotiopsis linearis* (MFLUCC 12-0271) and *P. trachycarpicola* (IFRDCC 2240).

Analysis 3: the combined sequence dataset of ITS, *tub2*, and *tef1-α* comprised 44 *Pseudopestalotiopsis* strains and two outgroup taxa, *Pestalotiopsis linearis* (MFLUCC 12-0271) and *P. trachycarpicola* (IFRDCC 2240) (**Figure 3**). The aligned dataset consisted of a total 1,848 characters with gaps (ITS: 1–550 bp, *tef1-α*: 551–1067 bp, *tub2*: 1068–1848 bp). The best-scoring RAxML tree was selected to represent the relationships among taxa, with a final likelihood value of −6,642.700192. The matrix contained 554 distinct alignment patterns, with 16.44% undetermined characters or gaps. Estimated base frequencies were as follows: A = 0.243417, C = 0.274291, G = 0.213172, and T = 0.269120; substitution rates were AC = 1.025029, AG = 2.296252, AT = 1.005669, CG = 0.871957, CT = 3.231208, and GT = 1.000000; gamma distribution shape parameter *α* = 0.306881, Tree-Length = 0.625052. For BI analysis, the best-fitting model of each locus and the final average standard deviation of split frequencies are shown in **Table 4**. Similar tree topologies were acquired by ML and BYPP analyses. The phylogenetic tree showed that *Pseudopestalotiopsis iteae* (SDBR-CMU514, SDBR-CMU519, SDBR-CMU520, SDBR-CMU521, SDBR-CMU522, SDBR-CMU523, and SDBR-CMU524) grouped with *Ps. theae* strain NTUCC 18-067 (70% ML and 0.92 BYPP) and formed a distinct subclade basal to the ex-epitype of *Ps. theae* (MFLUCC 12-0055) (**Figure 3**).

### 3.2 Taxonomy

**Figure 4:**
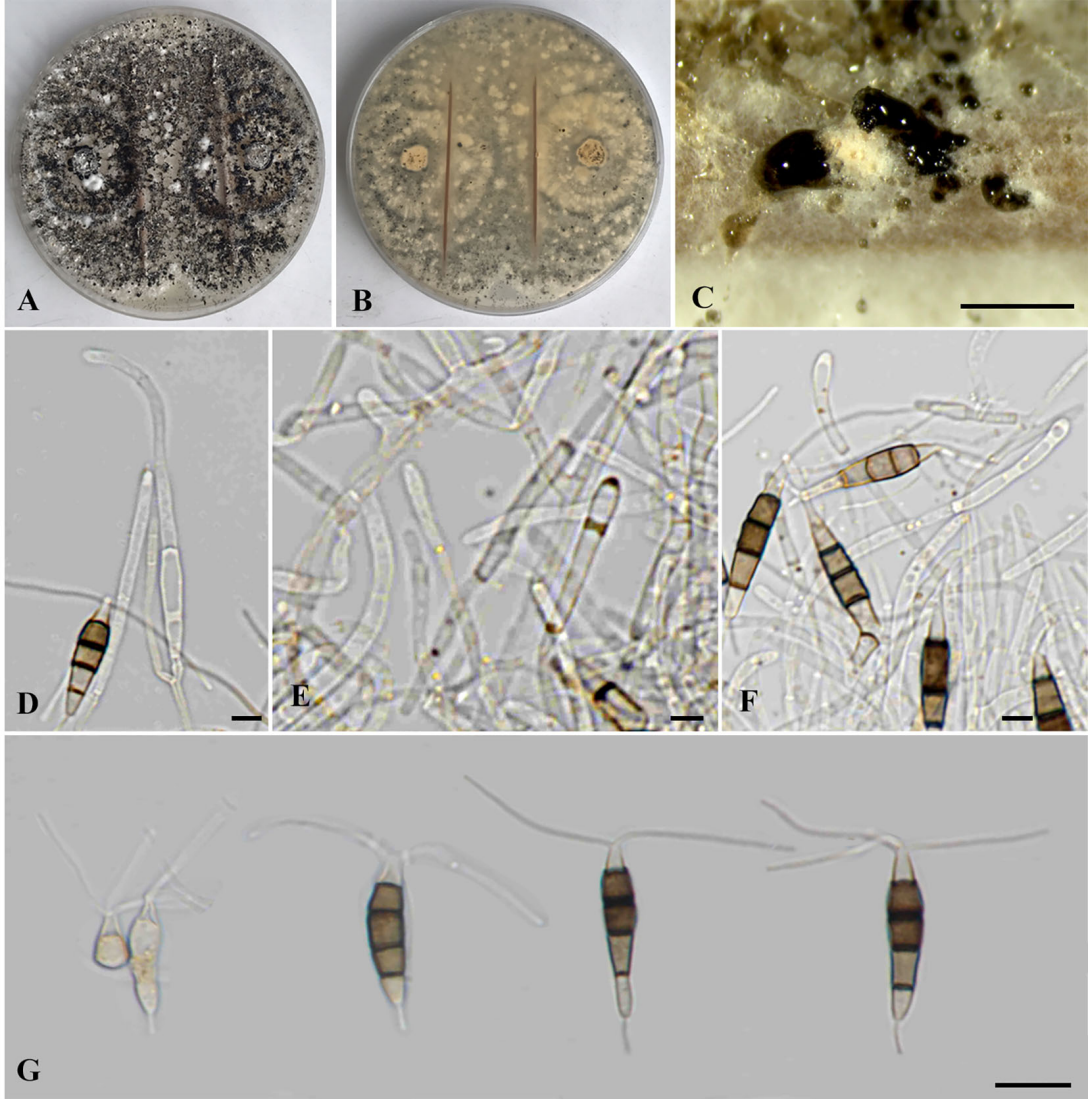
*Neopestalotiopsis chrysea* (SDBR-CMU516). **(A)** Surface of colonies on PDA. **(B)** Reverse of colonies on PDA. **(C)** Conidiomata and conidia masses. **(D–F)** Conidiophores, conidiogenous cells and conidia. **(G)** Conidia. Scale bars: **(B, C)** 500 μm, **(D–F)** 5 μm, and **(G)** 10 μm.

*Neopestalotiopsis chrysea* (Maharachch. & K.D. Hyde) Maharachch., K.D. Hyde & Crous, *Stud. Mycol.* 79: 138 (2014) (**Figure 4**).

≡ *Pestalotiopsis chrysea* Maharachch. and K.D. Hyde, *Fungal Divers.* 56(1): 107 (2012).

= *Neopestalotiopsis umbrinospora* (Maharachch. and K.D. Hyde) Maharachch., K.D. Hyde & Crous, *Stud. Mycol.* 79: 149 (2014).

Typification: CHINA, Guangxi Province, Shangsi, Shiwandashan, Wangle, dead leaves of unidentified plant, 2 January 1997, W.P. Wu (HMAS042855, holotype; MFLU 12-0411, isotype, ex-type culture NN042855 = MFLUCC 12-0261).

Description of the new collection: *Endophytic* on healthy roots of *Itea riparia*. Sexual morph: Undetermined. Asexual morph: *Conidiomata* up to 600 μm diameter, globose to subglobose, solitary to aggregated, black, semi-immersed on PDA, releasing globose, brown to black, slimy conidial masses. *Conidiophores* up to 65 μm long × 2–3 μm wide, hyaline, brown septa, branched. *Conidiogenous cells* 6–22 × 1.5–3 μm ( $\bar{x}$ = 15.5 × 2 μm, *n* = 10), discrete, holoblastic, subcylindrical to lageniform, hyaline, smooth-walled. *Conidia* 18.5–27.5 × 4–6 μm ( $\bar{x}$ = 22 × 5 μm, *n* = 30), fusiform to elongated fusiform, or ellipsoid, narrower towards the basal cells, straight to slightly curved, 4-septate; basal cell 3–7 μm long, subcylindrical to obconic, hyaline to pale brown, thin and smooth-walled; three median cells 12–16 μm long, sometimes hyaline to pale brown, one median cell when immature, pale brown to dark brown, with thick and darker brown septa, versicolored, second cell from base 4–4.5 μm, pale to light brown; third cell 4–4.5 μm, darker brown to olivaceous; fourth cell 4–4.5 μm, pale brown to olivaceous; apical cell 3–5 μm long, hyaline, obconic to conic; with two to three apical appendages, arising from the apical crest, filiform, unbranched, 24–30 μm long; with a single basal appendage, filiform, unbranched, centric, 3–6 μm long.

Culture characteristics: *Colonies* on PDA reached at 7 cm diameter after 5 days at 25°C, irregular form, raised elevation, crenate edge, whitish, with sparse aerial mycelium on surface, yellowish to pale brown in reverse. Sporulation on PDA at 25°C after 30 days, with numerous black fruiting bodies.

Material examined: THAILAND, Chiang Mai Province, Mae Rim District, on healthy roots of *Itea riparia* (*Iteaceae*), 10 July 2023, J. Monkai, IT107 (CMUB 40073), living culture, SDBR-CMU516 = KUNCC 24-18917, dried culture permanently preserved in a metabolically inactive state, CMUB 40073.

GenBank number: PQ521226 (ITS), PQ560699 (*tub2*), and PQ529177 (*tef1-α*).

Habitats and host: Dead and living tissue of *Carya illinoinensis*, *Itea riparia*, *Vaccinium ashei*, and unidentified plants (Maharachchikumbura et al., 2012; 2014; Shi et al., 2017; Wu et al., 2021; this study).

Distribution: China (Anhui, Fujian, Guangxi and Hunan Provinces) and Thailand (Maharachchikumbura et al., 2012; 2014; Shi et al., 2017; Wu et al., 2021; this study).

Notes: Based on the nucleotide BLAST search of ITS sequence, the new isolate of *Neopestalotiopsis chrysea* (SDBR-CMU516) showed the closest similarity with *Neopestalotiopsis* sp. 15 SSNM-2014 strain CBS 177.25 (100%), *Pestalotiopsis* sp. LH162 (100%), and *N. clavispora* strain YZM-1 (100%). Based on the nucleotide BLAST search of *tub2* sequence, *N. chrysea* (SDBR-CMU516) showed the closest similarity with *Neopestalotiopsis* sp. strain LC3480 (99.35%), *N. asiatica* isolate JGGH5 (99.35%), and *N. chrysea* isolate LSCKS81 (99.35%). Based on the nucleotide BLAST search of *tef1-α* sequence, *N. chrysea* (SDBR-CMU516) showed the closest similarity with *Pestalotiopsis chrysea* strain MFLUCC12-0261 (99.59%), *Neopestalotiopsis* sp. strain LC3480 (99.59%), and *N. chrysea* strain FZXM038 (99.38%).

Our new strain (SDBR-CMU516) was phylogenetically close to *Neopestalotiopsis chrysea* and *N. umbrinospora* (**Figure 1**). The nucleotide comparison between our new strain (SDBR-CMU516) and *N. chrysea* (MFLUCC 12-0261, ex-type) showed 0.9% (4/430) and 0.4% (2/487) bp difference in *tub2* and *tef1-α* (whereas those in ITS are identical). The nucleotide comparison between our new strain (SDBR-CMU516) and *N. umbrinospora* (MFLUCC 12-0285, ex-type) showed 0.9% (4/430) and 0.8% (4/487) bp difference in *tub2* and *tef1-α* (whereas those in ITS are identical). The nucleotide comparison between *N. chrysea* (MFLUCC 12-0261, ex-type) and *N. umbrinospora* (MFLUCC 12-0285, ex-type) showed 0.4% (2/487) in *tef1-α* (whereas those in ITS and *tub2* are identical).

*Pestalotiopsis chrysea* and *P. umberspora* were initially introduced by Maharachchikumbura et al. (2012), which transferred to *Neopestalotiopsis chrysea* and *N. umbrinospora* based on morphology and molecular phylogeny (Maharachchikumbura et al., 2014). *Neopestalotiopsis chrysea* was described by their distinct yellowish conidiogenous cells, conidia, and colony (Maharachchikumbura et al., 2012; 2014), whereas *N. umbrinospora* was characterized by its umber color of the median cells of the conidia (Maharachchikumbura et al., 2012; 2014). Both species were isolated from dead plant materials from Guangxi and Hunan China (Maharachchikumbura et al., 2012; 2014). Although there are only few base-pair differences between *N. umbrinospora* and *N. chrysea*, Maharachchikumbura et al. (2012; 2014) treated *N. umbrinospora* differently from the latter species due to its umber color and relatively wider conidia (9–25 × 6–8 μm vs. 20–24 × 5.5–7 μm), whereas our new strain (SDBR-CMU516) shares similar conidial shapes, color, and size to *N. chrysea* in producing darker brown to olivaceous conidia (18.5–27.5 × 4–6 μm vs. 20–24 × 5.5–7 μm) with three-tubular apical appendages (24–30 μm vs. 22–30 μm) (Maharachchikumbura et al., 2012; 2014). Therefore, we identified this strain as a new host record of *N. chrysea* on *Itea* based on morphological and phylogenetic lines of evidence. Furthermore, *N. umbrinospora* is synonymized under *N. chrysea* herein due to their conspecific relationship based on phylogenetic evidence coupled with the nucleotide pairwise comparison of the sufficient genes.

**Figure 5:**
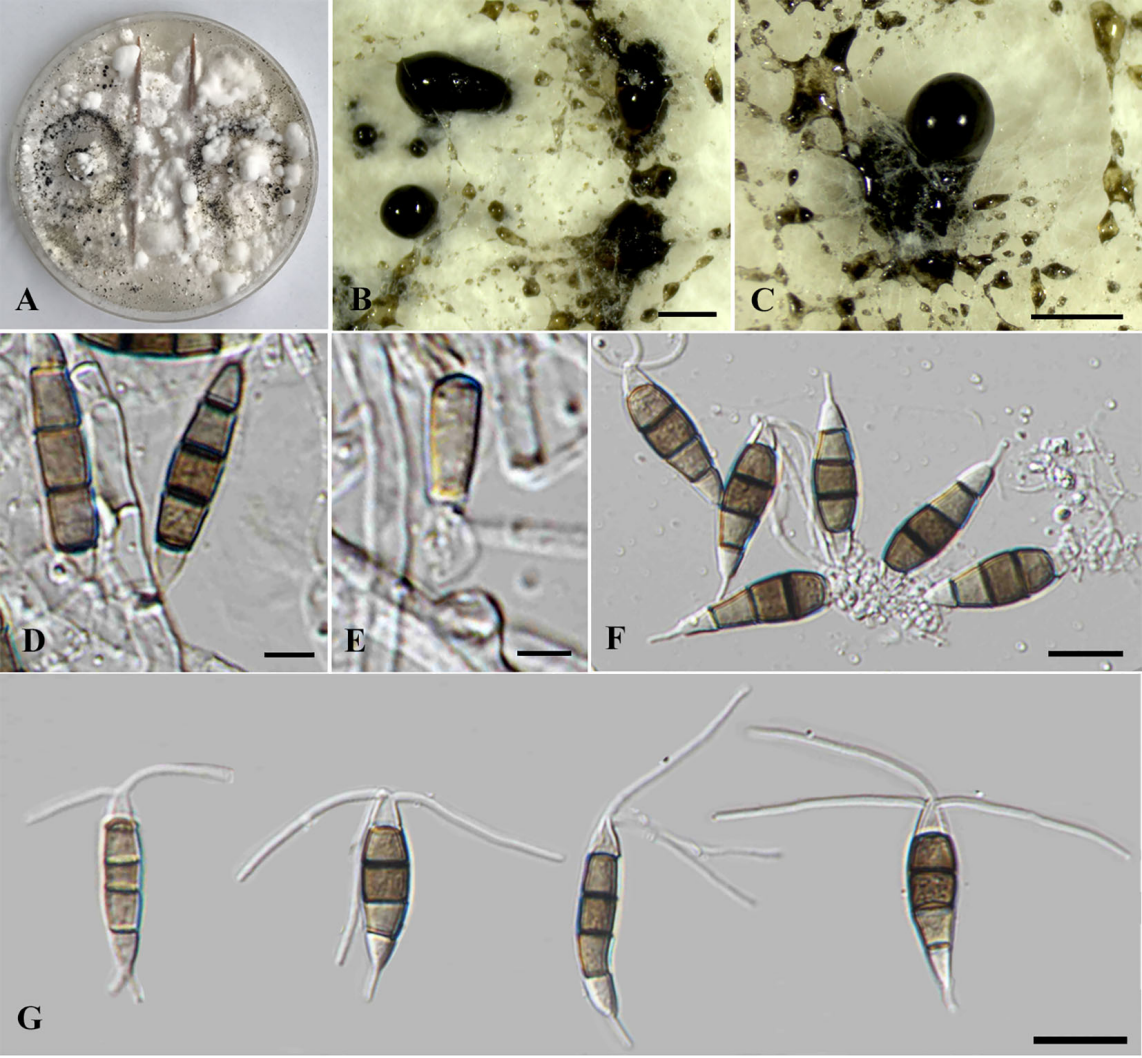
*Neopestalotiopsis haikouensis* (SDBR-CMU517). **(A)** Surface of colonies on PDA. **(B, C)** Conidiomata and conidia masses. **(D–F)** Conidiophores, conidiogenous cells, and conidia. **(G)** Conidia. Scale bars: **(B, C)** 500 μm, **(D–F)** 5 μm, and **(G)** 10 μm.

*Neopestalotiopsis haikouensis* Z.X. Zhang, J.W. Xia, and X.G. Zhang, *MycoKeys* 88: 181 (2022) (**Figure 5**).

= *Neopestalotiopsis cercidicola* W.S. Zhang and X.L. Fan, *J. Fungi* 10: 475 (2024).

= *Neopestalotiopsis terricola* W.L. Li and Jian K. Liu, *J. Fungi* 8: 1175 (2022).

Typification: CHINA, Hainan Province, Haikou City: East Harbour National Nature Reserve, on diseased leaves of *Ilex chinensis*, 23 May 2021, Z.X. Zhang (HSAUP212271, holotype), ex-type living culture: SAUCC212271.

Description of the new collection: *Endophytic* on healthy stems of *Itea riparia*. Sexual morph: Undetermined. Asexual morph: *Conidiomata* up to 500 μm diameter, pycnidial, globose to subglobose, solitary to aggregated, black, semi-immersed on PDA, releasing globose, brown to black, slimy conidial masses. *Conidiophores* up to 140 μm long × 2–3.5 μm wide, hyaline, dark brown septa, branched, sometimes giving rise to conidia. *Conidiogenous cells* 4–8.5 × 2.5–6 μm ( $\bar{x}$ = 7 × 4 μm, *n* = 10), discrete, holoblastic, subcylindrical to ampulliform, hyaline, smooth-walled. *Conidia* 20–30 × 5–7 μm ( $\bar{x}$ = 25 × 6 μm, *n* = 30), fusiform to elongated fusiform, or ellipsoid, sometimes slightly wider in the upper median cells, straight to slightly curved, 4-septate; basal cell 1–6 μm long, obconic to conic, hyaline, thin and smooth-walled; three median cells 13–18.5 μm long, pale brown when immature, pale brown to dark brown, darker at septa, versicolored, verruculose, second cell from base 4–6 μm, pale brown; third cell 4.5–5.5 μm, dark brown to olivaceous; fourth cell 3.5–6 μm, pale brown to olivaceous; apical cell 3.5–5 μm long, hyaline, subcylindrical to obconic; with two to three apical appendages (mostly three), arising from the apical crest, filiform, unbranched, 25–32.5 μm long; with a single basal appendages, filiform, unbranched, centric, 3–12 μm long.

Culture characteristics: *Colonies* on PDA reached at 7 cm diameter after 5 days at 25°C irregular form, raised elevation, crenate edge, whitish, cottony, with dense aerial mycelium on surface. Sporulation on PDA at 25°C after 30 days, with numerous black fruiting bodies.

Material examined: THAILAND, Chiang Mai Province, Mae Rim District, on living stems of *Itea riparia* (*Iteaceae*), 10 July 2023, J. Monkai, IT92 (CMUB40074), living culture, SDBR-CMU517 = KUNCC 24-18918, dried culture permanently preserved in a metabolically inactive state, CMUB40074.

GenBank number: PQ521227 (ITS), PQ560700 (*tub2*), and PQ529178 (*tef1-α*).

Habitats and host: Dead and living tissue of *Castanea mollissima*, *Cercis chinensis*, *Ilex chinensis*, *Itea riparia*, *Paeonia suffruticosa*, and *Olea europaea* (Jiang et al., 2021; Li et al., 2022; Zhang et al., 2022; 2024; this study).

Distribution: China (Hainan, Sichuan and Yunnan Provinces) and Thailand (Jiang et al., 2021; Li et al., 2022; Zhang et al., 2022; 2024; this study).

Notes: Based on the nucleotide BLAST search of ITS sequence, the new isolate of *Neopestalotiopsis haikouensis* (SDBR-CMU517) showed the closest similarity with *N. saprophytica* (100%), *Hymenopleella hippophaeicola* isolate SF134_3_1 (100%), and *Pestalotiopsis microspora* isolate WH55 (100%). Based on the nucleotide BLAST search of *tub2* sequence, *N. haikouensis* (SDBR-CMU517) showed the closest similarity with *Neopestalotiopsis* sp. strain PP026 (99.73%), *N. clavispora* isolate MCH21 (99.73%), and *N. clavispora* isolate SGP37 (99.73%). Based on the nucleotide BLAST search of *tef1-α* sequence, *N. haikouensis* (SDBR-CMU517) showed the closest similarity with *N. protearum* strain GBLZ16PE-007 (99.8%), *N. protearum* strain GUCC 23-0329 (99.8%), and *Neopestalotiopsis* sp. strain LC2945 (99.8%).

**Figure 6:**
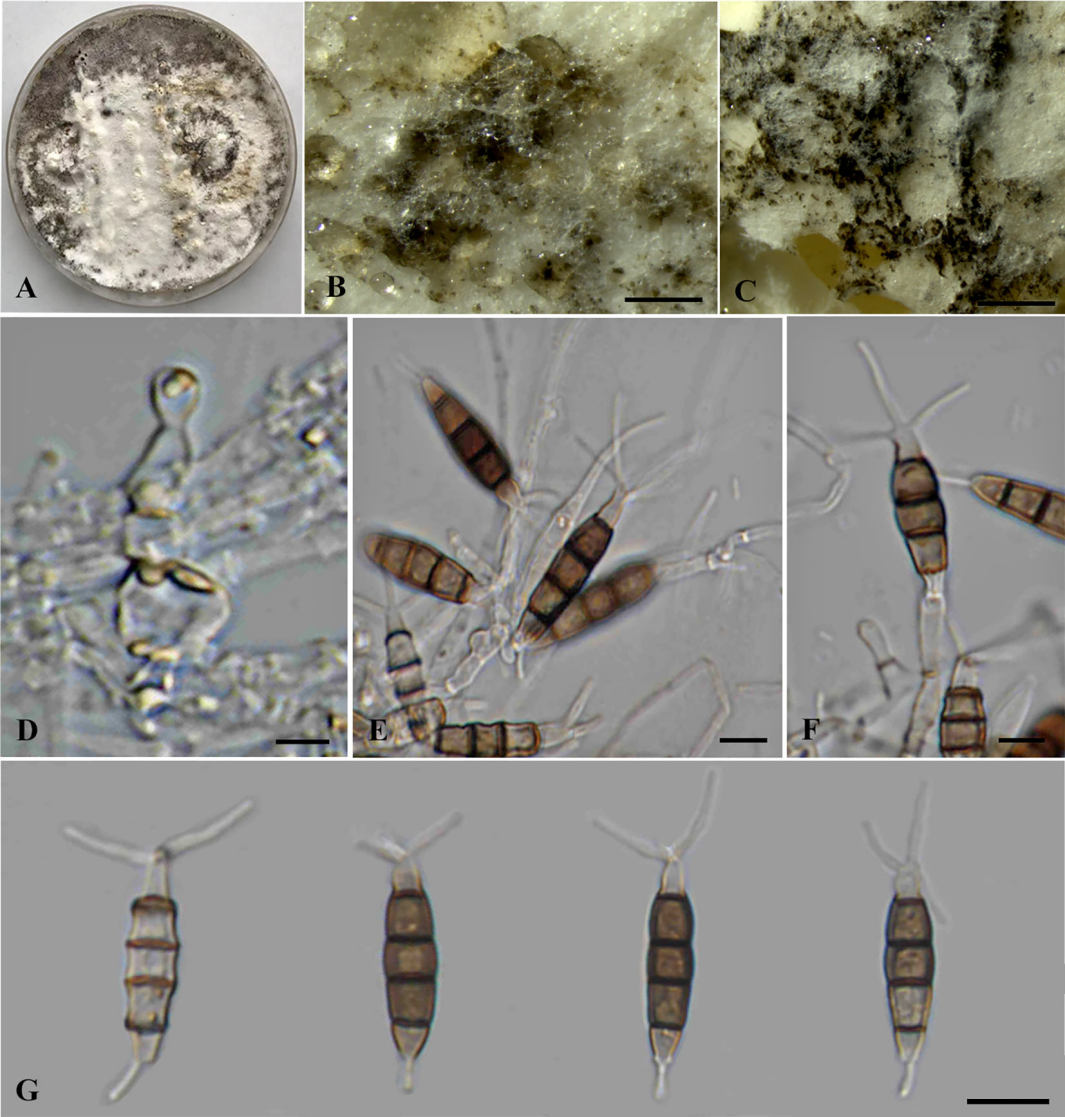
*Neopestalotiopsis iteae* (SDBR-CMU515, ex-type). **(A)** Surface of colonies on PDA. **(B, C)** Conidiomata. **(D–F)** Conidiophores, conidiogenous cells, and conidia. **(G)** Conidia. Scale bars: **(B, C)** 500 μm, **(D–F)** 5 μm, and **(G)** 10 μm.

In the phylogenetic analyses (**Figure 1**), our new strain (SDBR-CMU517) clustered with *Neopestalotiopsis cercidicola*, *N. haikouensis*, and *N. terricola*. The nucleotide comparison among our new strain (SDBR-CMU517) and the ex-type strains of *N. cercidicola*, *N. haikouensis*, and *N. terricola* indicated low nucleotide difference (less than 1%) (**Table 5**). Zhang et al. (2022) introduced *N. haikouensis* as a leaf spot disease of *Ilex chinensis*. Lately, *N. terricola* and *N. cercidicola* were isolated as pathogens of *Paeonia suffruticosa* (Li et al., 2022) and *Cercis chinensis* (Zhang et al., 2024), respectively. Based on morphology, our new strain (SDBR-CMU517) shares similar conidial color (pale brown) and size (20–30 × 5–7 μm) to *N. haikouensis* (16–22 × 4.5–7 μm) (Zhang et al., 2022) and *N. cercidicola* (17.5–23.5 × 5.5–8.5 μm) (Zhang et al., 2024) (**Table 6**), whereas the conidia of *N. terricola* is wider and darker in the middle cell (20–23 × 8–9.5 μm), compared to others (Li et al., 2022) (**Table 6**). Li et al. (2022) noted the variation of conidial color and size among strains of *N. terricola* depending on host substrates. The ex-type strain of *N. terricola* (CGMCC3.23553), which was isolated from diseased leaves of *Paeonia suffruticosa*, had darker and relatively wider conidia than other strains including the strain UESTCC 22.0034, isolated from infected olive leaves (see **Figures 6** and 9 in Li et al., 2022), and two strains (CFCC 54337 and CFCC 54340) recovered from diseased leaves of *Castanea mollissima* (see Figure 17 in Jiang et al., 2021) (**Table 6**). Moreover, we could detect the varieties of conidial shape and size in our samples, which is shown in **Figure 5G**. Therefore, because of the morphological and phylogenetic consistencies, we synonymized *N. terricola* and *N. cercidicola* under *N. haikouensis* following the prior publication. Likewise, our new strain is reported as a new host record of *N. haikouensis* on *Itea* and it is also a new geographical record in Thailand.

**Table 5:** Nucleotide differences among the closely related strains of *Neopestalotiopsis haikouensis*.

Compared strains	Gene region/locus		
	ITS	*tub2*	*tef1-α*
The new strain (SDBR-CMU517) vs. *N. haikouensis* (SAUCC 212271)	0.2% (1/503)	0.3% (2/747)	Identical
The new strain (SDBR-CMU517) vs. *N. haikouensis* (=*N. terricola*) (CGMCC 3.23553)	Identical	0.3% (2/727)	Identical
The new strain (SDBR-CMU517) vs. *N. haikouensis* (=*N. cercidicola*) (CFCC 7063)	0.4% (2/494)	0.3% (2/632)	0.5% (2/444)
*N. haikouensis* (SAUCC 212271) vs. *N. haikouensis* (=*N. terricola*) (CGMCC 3.23553)	Identical	0.1% (1/735)	Identical
*N. haikouensis* (SAUCC 212271) vs. *N. haikouensis* (=*N. cercidicola*) (CFCC 7063)	0.4% (2/489)	0.3% (2/632)	0.2% (1/439)
*N. terricola* (CGMCC 3.23553) vs. *N. haikouensis* (=*N. cercidicola*) (CFCC 7063)	0.2% (1/485)	0.5% (2/434)	0.3% (2/712)

The data are represented by the number of different nucleotides/numbers of all nucleotides (% base-pair difference).

**Table 6:** The comparison of conidial morphology and host between the closely related strains of *Neopestalotiopsis haikouensis*.

Strains	Conidial size	Host	References
**The new strain (SDBR-CMU517)**	**20–30 × 5.3–7 μm (25 × 6 μm)**	**Healthy stems of *Itea riparia***	**This study**
*N. haikouensis* (SAUCC 212271)	16.0–22.0 × 4.5–7.0 μm (20.0 × 5.5 μm)	Diseased leaves of *Ilex chinensis*	Zhang et al. (2022)
*N. haikouensis* (=*N. terricola*) (CGMCC 3.23553)	20–23 × 8–9.5 μm (21.5 × 8.5 μm)	Diseased branch of *Paeonia suffruticosa* and diseased leaf of *Olea europaea*	Li et al. (2022)
*N. haikouensis* (=*Neopestalotiopsis* sp.1) (CFCC 54337)	19.1–24.7 × 5.4–8.6 μm (21.6 × 6.7 μm)	Leaf spots of *Castanea mollissima*	Jiang et al. (2021)
*N. haikouensis* (=*Neopestalotiopsis* sp.2) (CFCC 54340)	21.4–26.2 × 5.1–8.7 μm (23.6 × 7 μm)	Leaf spots of *Castanea mollissima*	Jiang et al. (2021)
*N. haikouensis* (=*N. cercidicola*) (CFCC 7063)	17.5–23.5 *×* 5.5–8.5 μm (20.7 × 6.9 μm)	Leaf spots of *Cercis chinensis*	Zhang et al. (2024)

The new strain obtained in this study is indicated in black bold.

*Neopestalotiopsis iteae* Monkai, Phookamsak, Bhat & S. Lumyong, sp. nov. (**Figure 6**).

Index Fungorum number: IF902438.

*Etymology*: Refers to the host genus, *Itea* from which the holotype was collected.

*Endophytic* on healthy stems of *Itea japonica*. Sexual morph: Undetermined. Asexual morph: *Conidiomata* up to 400 μm diameter, globose to subglobose, solitary to aggregated, black, raising above surface of PDA. *Conidiophores* up to 50 μm long × 2–3 μm wide, hyaline, dark brown septa, branched. *Conidiogenous cells* 4–9.5 × 2.5–4 μm ( $\bar{x}$ = 6 × 3 μm, *n* = 10), discrete, holoblastic, subcylindrical to ampulliform, hyaline, smooth-walled. *Conidia* 17.5–26 × 4.5–6.5 μm ( $\bar{x}$ = 22.5 × 5.5 μm, *n* = 30), fusiform, ellipsoid, straight, 4-septate; basal cell 4–5.5 μm long, obconic to conic, hyaline, thin and smooth-walled; three median cells 12–17.5 μm long, initially hyaline with brown septa, becoming brown to pale brown, with darker brown septa, slightly constricted at septa, with second cell from base 4–6.5 μm, pale brown to olivaceous; third cell 3.5–6 μm, brown to olivaceous; fourth cell 4–6 μm, brown to olivaceous; apical cell 4.5–6 μm long, hyaline, subcylindrical to obconic; with two to three apical appendages, not arising from the apical crest, but each inserted at a different locus in the upper half of the apical cell, filiform, unbranched, 8.5–14 μm long; with a single basal appendages, filiform, unbranched, 4.5–6 μm long.

Culture characteristics: *Colonies* on PDA reached at 7 cm diameter after 5 days at 25°C, circular form, flat elevation, undulage edge, whitish to gray, with dense aerial mycelium on surface. Sporulation on PDA at 25°C after 30 days, with numerous black fruiting bodies.

Material examined: THAILAND, Chiang Mai Province, Hang Dong District, on living stems of *Itea japonica* (*Iteaceae*), 7 February 2023, J. Monkai, IT41 (CMUB40072, holotype), ex-type living culture, SDBR-CMU515 = KUNCC 24-18919, dried culture permanently preserved in a metabolically inactive state, CMUB40072.

GenBank number: PQ521228 (ITS), PQ560701 (*tub2*), and PQ529179 (*tef1-α*).

Notes: Based on the nucleotide BLAST search of ITS sequence, *Neopestalotiopsis iteae* sp. nov. (SDBR-CMU515) showed the closest similarity with *N. clavispora* isolate MI003 (100%), *N. saprophytica* (100%), and *Pestalotiopsis maculans* isolate RM1.18A.01 (100%). Based on the nucleotide BLAST search of *tub2* sequence, *N. iteae* (SDBR-CMU515) showed the closest similarity with *N. piceana* (99.12%), *N. piceana* strain CBS 225.30 (99.12%), and *Neopestalotiopsis* sp. strain PP026 (99.12%). Based on the nucleotide BLAST search of *tef1-α* sequence, *N. iteae* (SDBR-CMU515) showed the closest similarity with *Neopestalotiopsis* sp. isolate YJ11-0708 (98.57%), *N. clavispora* isolate SGP35 (98.57%), and *N. clavispora* isolate MCH27 (98.57%).

Phylogenetically, *Neopestalotiopsis iteae* formed a stable clade sister to *N. cocoës* (0.91 BYPP; **Figure 1**). The nucleotide comparison between *N. iteae* (SDBR-CMU515) and *N. cocoës* (MFLUCC 15-0152, ex-type) showed 0.4% (2/520) and 2.4% (6/253) bp differences in ITS and *tef1-α* (where *tub2* sequence data of *N. cocoës* were not available for comparison). It should be noted that the *tef1-α* sequence data of *N. cocoës* are very short (approximately 200–300 bp). The low support between *N. iteae* and *N. cocoës* might be coming from the uncompleted sequence data of *N. cocoës*. Morphologically, *N. iteae* can be distinguished from *N. cocoës* in having longer and narrower conidia (17.5–26 × 4.5–6.5 μm vs. 19–22.5 × 7.5–9.5 μm) and slightly constricted septa (Hyde et al., 2016). Based on the lines of evidence in morphology and phylogeny coupled with a significant nucleotide pairwise difference of *tef1-α* sequence, *N. iteae* was introduced as a novel species.

**Figure 7:**
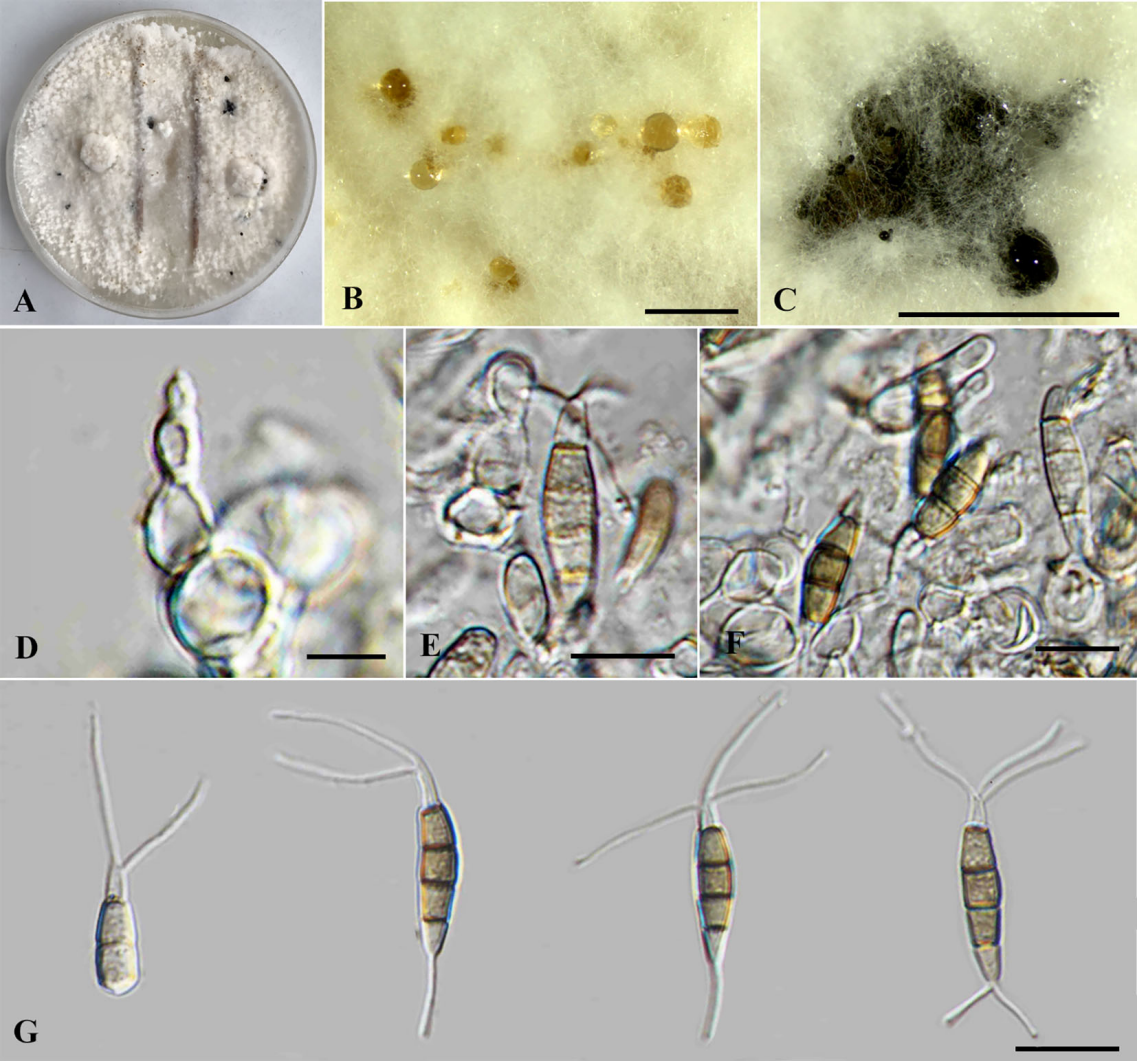
*Pestalotiopsis jinchanghensis* (SDBR-CMU518). **(A)** Surface of colonies on PDA. **(B, C)** Conidiomata and conidia masses. **(D–F)** Conidiogenous cells with attached conidia. **(G)** Conidia. Scale bars: **(B, C)** 500 μm and **(D–G)** 10 μm.

*Pestalotiopsis jinchanghensis* Liu, Hou, Raza and Cai, *Scientific Reports* 7 (no. 866): 8 (2017) (**Figure 7**).

= *Pestalotiopsis zhaoqingensis* H.J. Zhao and W. Dong, *Mycosphere* 14(1): 2238 (2023).

Typification: CHINA, Yunnan Province, Xishuangbanna, Jinchanghe, on leaves of *Camellia sinensis*, 20 April 2015, F. Liu, HMAS 247061 (holotype), ex-type living culture CGMCC 3.18158 (= LC663*6*).

Description of the new collection: *Endophytic* on healthy stems of *Itea riparia*. Sexual morph: Undetermined. Asexual morph: *Conidiomata* up to 600 μm diameter, pycnidial, globose to subglobose, solitary, black, semi-immersed on PDA, releasing globose, brown to black, slimy conidial masses. *Conidiophores* often reduced to conidiogenous cells. *Conidiogenous cells* 4–10 × 2.5–8.5 μm ( $\bar{x}$ = 7.5 × 6.5 μm, *n* = 10), discrete, holoblastic, ampulliform to lageniform, hyaline, smooth-walled. *Conidia* 19–27 × 4–6 μm ( $\bar{x}$ = 23.5 × 5 μm, *n* = 30), fusiform to elongated fusiform, straight to slightly curved, 4-septate; basal cell 3.5–4.5 μm long, obconic to conic, hyaline, thin and smooth-walled; three median cells 12–16.5 μm long, sometimes hyaline, two median cells when immature, brown to olivaceous, septa and periclinal walls darker than the rest of the cell, concolorous, wall rugose, second cell from base 4–5 μm; third cell 3.5–5.5 μm; fourth cell 4–5.5 μm; apical cell 3.5–4.5 μm long, hyaline, subcylindrical to obconic; with two to three tubular apical appendages, arising from the apical crest, filiform, unbranched, 14–27 μm long; with 1(–2) tubular basal appendages, filiform, unbranched, centric, 5–11.5 μm long.

Culture characteristics: *Colonies* on PDA reached at 7 cm diameter after 5 days at 25°C, irregular form, raised elevation, crenate edge, whitish, cottony, with dense aerial mycelium on surface. Sporulation on PDA at 25°C after 30 days, with few black fruiting bodies.

Material examined: THAILAND, Chiang Mai Province, Mae Rim District, on living stems of *Itea riparia* (*Iteaceae*), 10 July 2023, J. Monkai, IT86 (CMUB40075), living culture, SDBR-CMU518 = KUNCC 24-18920), dried culture permanently preserved in a metabolically inactive state, CMUB40075.

GenBank number: PQ521229 (ITS), PQ560702 (*tub2*), and PQ529180 (*tef1-α*).

Habitats and host: Dead and living tissue of *Camellia sinensis*, *Itea riparia*, and unidentified plants (Liu et al., 2017; Dong et al., 2023; this study).

Distribution: China (Yunnan and Guangdong Provinces) and Thailand (Liu et al., 2017; Dong et al., 2023; this study).

Notes: Based on the nucleotide BLAST search of ITS sequence, the new isolate of *Pestalotiopsis jinchanghensis* (SDBR-CMU518) showed the closest similarity with *Pestalotiopsis* sp. NJ-2022e strain SAUCC230044 (99.83%), *Pestalotiopsis* sp. strain JMB08_3B2 (99.67%), and *P. malayana* strain SAUCC230483 (99.66%). Based on the nucleotide BLAST search of *tub2* sequence, *P. jinchanghensis* (SDBR-CMU518) showed the closest similarity with *P. zhaoqingensis* strain ZHKUCC 23-0825 (99.87%), *P. jinchanghensis* strain LC8190 (99.87%), and *P. jinchanghensis* strain LC8191 (99.87%). Based on the nucleotide BLAST search of *tef1-α* sequence, *P. jinchanghensis* (SDBR-CMU518) showed the closest similarity with *P. jinchanghensis* strain LC8191 (99.79%), *P. jinchanghensis* strain LC6636 (99.79%), and *P. jinchanghensis* strain LC8190 (99.79%).

The combined phylogenetic tree indicated that our new strain (SDBR-CMU518) clustered in the same clade with *Pestalotiopsis jinchanghensis* and *P. zhaoqingensis* with 86% ML, 1.00 BYPP statistical support (**Figure 2**). The nucleotide comparison between our new strain (SDBR-CMU518) and *P. jinchanghensis* (CGMCC 3.18158, ex-type) showed 0.2% (1/475) base-pair difference in *tef1-α* (whereas those in ITS and *tub2* are identical). The nucleotide comparison between our new strain (SDBR-CMU518) and *P. zhaoqingensis* (ZHKUCC 23-0825, ex-type) showed 1% (6/563), 0.1% (1/127), and 0.2% (1/447) bp differences in ITS, *tef1-α*, and *tub2*. In addition, the nucleotide comparison between *P. jinchanghensis* (CGMCC 3.18158, ex-type) and *P. zhaoqingensis* (ZHKUCC 23-0825, ex-type) showed 1.2% (6/500) and 0.2% (1/449) bp differences in ITS and *tef1-α* (whereas those in *tub2* are identical). *Pestalotiopsis jinchanghensis* was identified as a disease on *Camellia sinensis* (Liu et al., 2017), and *P. zhaoqingensis* was isolated from dead unknown plants in China (Dong et al., 2023). Although there were relatively low nucleotide variations between *P. jinchanghensis* and *P. zhaoqingensis*, Dong et al. (2023) justified *P. zhaoqingensis* as distinct species based on morphology, wherein it produced comparatively shorter conidia (17–24 × 4–8 μm vs. 22–32 × 5.5–8.5 μm) and branched apical appendages. Our new strain (SDBR-CMU518) mostly resembles *P. jinchanghensis* in having unbranched apical appendages (Liu et al., 2017). However, the conidial size of our new isolate showed to be overlapping with both *P. jinchanghensis* and *P. zhaoqingensis* (19–27 × 4–6 μm vs. 22–32 × 5.5–8.5 μm vs. 17–24 × 4–8 μm). Based on morphological and phylogenetic lines of evidence as well as an identical nucleotide pairwise, we treated the new strain as a new host record of *P. jinchanghensis* on *Itea* and the species is reported from Thailand for the first time, while *P. zhaoqingensis* is synonymized under *P. jinchanghensis* herein.

**Figure 8:**
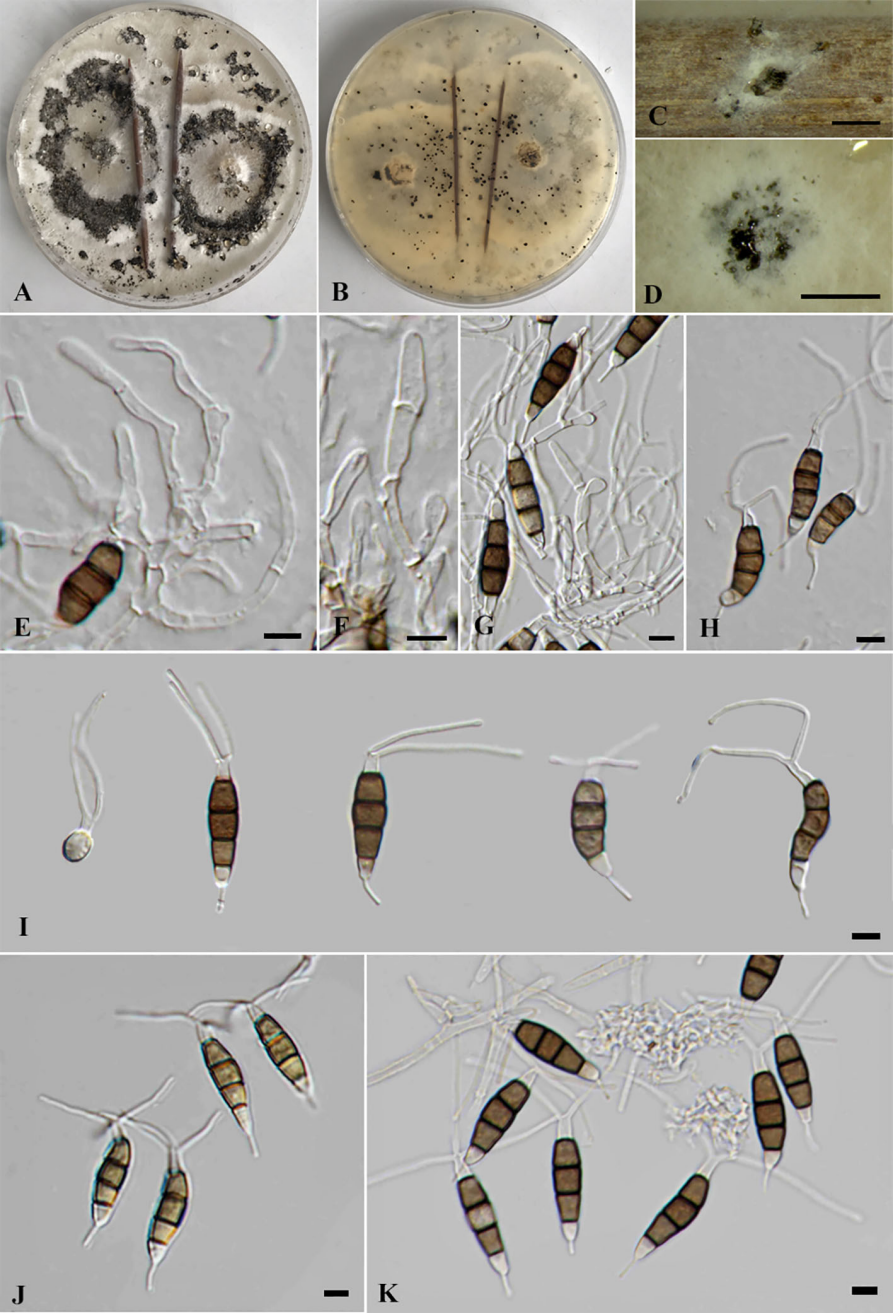
*Pseudopestalotiopsis iteae* [SDBR-CMU514, ex-type **(A–I)**], SDBR-CMU523 **(J)**, and SDBR-CMU524 **(K)**. **(A)** Surface of colonies on PDA. **(B)** Reverse of colonies on PDA. **(C, D)** Conidiomata and conidia masses. **(E–G)** Conidiophores, conidiogenous cells, and conidia. **(H–K)** Conidia. Scale bars: **(C, D)** 500 μm and **(E–K)** 5 μm.

*Pseudopestalotiopsis iteae* Monkai, Phookamsak, Bhat & S. Lumyong, sp. nov. (**Figure 8**).

Index Fungorum number: IF902439.

*Etymology*: Refers to the host genus from which the holotype was collected, *Itea*.

*Endophytic* on healthy leaves and roots of *Itea riparia*. Sexual morph: Undetermined. Asexual morph: *Conidiomata* up to 500 μm diameter, globose to subglobose, solitary to aggregated, black, semi-immersed on PDA, releasing globose, brown to black, slimy conidial masses. *Conidiophores* 34–52 × 1.5–3 μm ( $\bar{x}$ = 43 × 2 μm, *n* = 10), hyaline, septate, branched. *Conidiogenous cells* 9.5–16.5 × 2–4 μm ( $\bar{x}$ = 12.5 × 3 μm, *n* = 10), discrete, holoblastic, subcylindrical to ampulliform, hyaline, smooth-walled. *Conidia* 20–27 × 4.5–7 μm ( $\bar{x}$ = 24 × 6 μm, *n* = 30), fusiform to ellipsoid, straight to slightly curved in C-form or S-form, 4-septate; basal cell 3–4.5 μm long, obconic to conic, hyaline, thin and smooth-walled; three median cells 12–17 μm long, sometimes hyaline, one to three median cells when immature, brown to dark brown, with thick verruculose walls, constricted at septa, septa and periclinal walls darker than the rest of the cell, concolorous, second cell from base 4–5.5 μm; third cell 4–5.5 μm; fourth cell 4–6.5 μm; apical cell 4–5.5 μm long, hyaline, subcylindrical to obconic; with two to three tubular apical appendages (mostly three), arising from the upper portion of apical cell, filiform, unbranched, knobbed, 18–21 μm long; with a single basal appendages (mostly one), filiform, unbranched, slightly knobbed, 4–7 μm long.

Culture characteristics: *Colonies* on PDA reached 7 cm diameter after 5 days at 25°C, irregular form, umbonate elevation, crenate edge, whitish, cottony, with dense aerial mycelium on surface, pale brown to white in reverse, Sporulation on PDA at 25°C after 30 days, with numerous black fruiting bodies.

Material examined: THAILAND, Chiang Mai Province, Mae Rim District, on living leaves of *Itea riparia* (*Iteaceae*), 10 July 2023, J. Monkai, IT48 (CMUB40071, holotype), ex-type living culture, SDBR-CMU514 = KUNCC24-18921, dried culture permanently preserved in a metabolically inactive state, CMUB40071; ibid., on living leaves of *Itea riparia* (*Iteaceae*), 10 July 2023, J. Monkai, IT49, living culture, SDBR-CMU519 = KUNCC24-18922; ibid., on living leaves of *Itea riparia* (*Iteaceae*), 10 July 2023, J. Monkai, IT50, living culture, SDBR-CMU520 = KUNCC24-18923; ibid., on living leaves of *Itea riparia* (*Iteaceae*), 10 July 2023, J. Monkai, IT61, living culture, SDBR-CMU521 = KUNCC24-18924; ibid., on living leaves of *Itea riparia* (*Iteaceae*), 10 July 2023, J. Monkai, IT62, living culture, SDBR-CMU522 = KUNCC24-18925; ibid., on living leaves of *Itea riparia* (*Iteaceae*), 10 July 2023, J. Monkai, IT73, living culture, SDBR-CMU523 = KUNCC24-18926; ibid., on living roots of *Itea riparia* (*Iteaceae*), 10 July 2023, J. Monkai, IT99, living culture, SDBR-CMU524 = KUNCC24-18927.

GenBank number: SDBR-CMU514; PQ521230 (ITS), PQ560703 (*tub2*), PQ529181 (*tef1-α*), SDBR-CMU519; PQ521231 (ITS), PQ560704 (*tub2*), PQ529182 (*tef1-α*), SDBR-CMU520; PQ521232 (ITS), PQ560705 (*tub2*), PQ529183 (*tef1-α*), SDBR-CMU521; PQ521233 (ITS), PQ560706 (*tub2*), PQ529184 (*tef1-α*), SDBR-CMU522; PQ521234 (ITS), PQ560707 (*tub2*), PQ529185 (*tef1-α*), SDBR-CMU523; PQ521235 (ITS), PQ560708 (*tub2*), PQ529186 (*tef1-α*), SDBR-CMU524; PQ521236 (ITS), PQ560709 (*tub2*), PQ529187 (*tef1-α*).

Notes: Based on the nucleotide BLAST search of ITS sequence, *Pseudopestalotiopsis iteae* sp. nov. (SDBR-CMU514) showed the closest similarity with *Pseudopestalotiopsis* sp. isolate ERS19.48.Le.D (99.63%), *Ps. theae* strain KU20018.104 (99.63%), and *Pestalotiopsis theae* isolate CPO/Pe (99.63%). Based on the nucleotide BLAST search of *tub2* sequence, *Ps. iteae* (SDBR-CMU514) showed the closest similarity with *Ps. theae* isolate TN07 (98.97%), *Ps. theae* strain GUCC 23-0406 (99.46%), and *Ps. theae* strain GUCC 23-0472 (99.86%). Based on the nucleotide BLAST search of *tef1-α* sequence, *Ps. iteae* (SDBR-CMU514) showed the closest similarity with *Ps. theae* strain GUCC 23-0406 (99.60%), *Pseudopestalotiopsis* sp. 2-KW-2016 strain: 10 (99.60%), and *Pseudopestalotiopsis* sp. 2-KW-2016 strain: 11 (99.39%).

Phylogenetic tree of combined ITS, *tub2*, and *tef1-α* sequence data revealed that *Pseudopestalotiopsis iteae* (SDBR-CMU514, SDBR-CMU519, SDBR-CMU520, SDBR-CMU521, SDBR-CMU522, SDBR-CMU523, and SDBR-CMU524) are clustered together with *Ps. theae* strain NTUCC 18-067, with significant support (70% ML, 0.92 BYPP), which are distant from the ex-epitype of *Ps. theae* (MFLUCC 12-0055) (**Figure 3**). The nucleotide comparison between *Ps. iteae* (SDBR-CMU514) and *Ps. theae* (MFLUCC 12-0055) showed 0.2% (1/491), 0.7% (3/426), and 1.8% (9/493) bp difference in ITS, *tub2*, and *tef1-α* (**Table 7**), respectively. Moreover, the nucleotide differences between the epitype of *Ps. theae* and other *Ps. iteae* strains are provided in **Table 7**. The morphological characteristics of *Ps. iteae* resemble those of *Ps. theae* in having brown concolorous median cells, constricted at septa with knobbed apical appendages (Maharachchchikumbura et al., 2013). However, *Ps. iteae* has narrower conidia (24 × 6 vs. 25.5 × 7.6 μm) and shorter apical appendages (18–21 μm vs. 22.5–31 μm) than *Ps. theae* (Maharachchikumbura et al., 2013). Thus, we introduced *Ps. iteae* as a new species based on the differences of nucleotide pairwise comparison of the sufficient gene (*tef1-α*) and morphological characteristics.

**Table 7:** Nucleotide differences between the epitype of *Pseudopestalotiopsis theae* and *Ps. iteae* strains.

Gene region/locus	The epitype of *Pseudopestalotiopsis theae* (MFLUCC 12-0055) compared with *Ps. iteae* strains							
	NTUCC 18-067	SDBR-CMU514 (ex-type)	SDBR-CMU519	SDBR-CMU520	SDBR-CMU521	SDBR-CMU522	SDBR-CMU523	SDBR-CMU524
ITS	0/489 (0%)	1/491 (0.2%)	1/491 (0.2%)	1/491 (0.2%)	1/491 (0.2%)	1/491 (0.2%)	1/491 (0.2%)	1/491 (0.2%)
*tub2*	2/424 (0.4%)	1/415 (0.2%)	1/413 (0.2%)	9/414 (2.1%)	1/404 (0.2%)	8/413 (1.9%)	3/402 (0.7%)	3/403 (0.7%)
*tef1-α*	11/473 (2.3%)	9/493 (1.8%)	11/509 (2.2%)	8/506 (1.6%)	8/502 (1.6%)	8/492 (1.6%)	9/493 (1.8%)	9/490 (1.8%)

The data represented by the number of different nucleotides/number of all nucleotides (% base-pair difference).

We noticed that the conidial characteristics varied between the strains of *Pseudopestalotiopsis iteae.* Compared with the type strain, strain SDBR-CMU523 produced shorter conidia with pale brown color (21 × 5 vs. 24 × 6 μm) and shorter appendages without knobbed tips (11–19 vs. 18–21 μm) (**Figure 8J**), while the strain SDBR-CMU524 has relatively longer conidia that are not constricted at septa (26 × 5 vs. 24 × 6 μm) and longer appendages (15–32 vs. 11–19 μm) than the type strain (**Figure 8K**). In phylogeny, strain SDBR-CMU524 formed a separated branch to other strains of *Ps. iteae* (**Figure 3**). However, there are too few base-pair differences in ITS, *tub2*, and *tef1-α* (less than 1%) between the ex-type strain of *Ps. iteae* (SDBR-CMU514) and strain SDBR-CMU524. The strain SDBR-CMU524 isolated from healthy roots and other strains isolated from healthy leaves of *Itea* spp. should also be noted. It is indicated that *Ps. iteae* has also high intraspecific variation, similar to *Ps. theae*.

*Pseudopestalotiopsis theae* (≡ *Pestalotia theae*) was originally isolated from *Camellia sinensis* in Taiwan, then Maharachchikumbura et al. (2013) designated the epitype of *Ps. theae* (MFLUCC 12-0055) from a sample collected from the same host in Thailand. Tsai et al. (2021) isolated a strain of *Ps. theae* (NTUCC 18-067) as a pathogen on *C. sinensis* in Taiwan, representing it as the reference strain. Our phylogeny demonstrated that this strain is not consistent with *Ps. theae* and their nucleotide variation of *tef1-α* is greater (2.3% bp difference) (**Table 7**), regarding it as *Ps. iteae*. Also, the ex-epitype strain of *Ps. theae* (MFLUCC 12-0055) is closely related with *Ps. theae* strain SC011 and *Ps. theae* strain MFLUCC 22-0128, which are from Thailand (Maharachchikumbura et al., 2013; 2014; Sun et al., 2023), and *Pseudopestalotiopsis* sp. strains NBRC112267 and NBRC112268, which are from Myanmar (Nozawa et al., 2017). Thus, these strains could be treated as *Ps. theae.*

Additionally, we examined the few nucleotide differences of *tub2* (less than 1%) between the ex-type of *Pseudopestalotiopsis iteae*, epitype of *Ps. theae*, and strain NTUCC 18-067. The *tub2* sequence data of those strains lacked the first 300–400 base-pair positions in the alignment of all *Pseudopestalotiopsis* taxa. This may be caused by the different primers used for PCR amplification of *tub2*, which are BT2A/BT2B in those studies (Maharachchikumbura et al., 2013; Tsai et al., 2021), while a primer pair, T1/BT2B, was used in our study and other studies (i.e. Norphanphoun et al., 2019; Sun et al., 2023). To clarify this issue, we then amplified our strains with the BT2A/BT2B, and the results did not show any significant nucleotide variation. Thus, the epitype of *Ps. theae*, strain NTUCC 18-067, and other related strains should be further analyzed for *tub2* using T1/BT2B primers as it allows longer base-pair length (up to 800 bp).

## 4 Discussion

The nomenclature and classification of pestalotioid fungi are remarkably challenging. At the generic level, phylogenetic analyses of combined ITS, *tub2*, and *tef1-α* provide sufficient distinction between *Neopestalotiopsis*, *Pestalotiopsis*, and *Pseudopestalotiopsis* (Maharachchikumbura et al., 2014; 2016; Norphanphoun et al., 2019; Sun et al., 2023), whereas the morphological circumscriptions of these genera remain inconclusive (Liu et al., 2017). At the species level, the combination of multi-locus phylogeny, morphology, and ecology has been employed for determining the interspecific variation (Maharachchikumbura et al., 2014; 2016; Tsai et al., 2018; 2021; Chen et al., 2018; Gu et al., 2022). According to their phenotypic plasticity that largely relies on environmental conditions and poor resolution derived from phylogenetic reconstruction, standard criteria for species delineation are still uncertain (Xiong et al., 2022; Guterres et al., 2023; Hsu et al., 2024). Recent studies recovered several undetermined pestalotioid taxa, most of which were not assigned a species name (Jiang et al., 2021; Tsai et al., 2021; Guterres et al., 2023; Hsu et al., 2024; Wang et al., 2024). During our taxonomic investigation of endophytic fungi associated with *Itea* spp., five *Pestalotiopsis*-like species were characterized using morphology, nucleotide pairwise comparison, and phylogenetic evidence. Two new species (*Neopestalotiopsis iteae* and *Pseudopestalotiopis iteae*) and three hitherto known species (*Pestalotiopsis jinchanghensis*, *N. chrysea*, and *N. haikouensis*) were reported for the first time on this host plant and were discussed accordingly.

The novel species, *Neopestalotiopsis iteae*, is phylogenetically adjacent to *N. cocoës* (**Figure 1**), but it produces constricted septate and longer conidia than the latter species. We also detected a significant nucleotide difference in *tef1-α* sequence and thus reported it as a new species. Our study pointed out that the phylogenetic placement of *N. iteae* received poor support value (**Figure 1**). This phenomenon was also observed in previous studies in which the phylograms of *Neopestalotiopsis* had unstable topology and mostly contained weak support (Liu et al., 2017; Tsai et al., 2021; Hsu et al., 2024), whereas a new record of *N. chrysea* was assigned based on the evidence of phylogenetic analyses and nucleotide differences of the sufficient genes. The novel strain (SDBR-CMU516) shared a close phylogenetic relationship with *N. chrysea* and *N. umbrinospora* (**Figure 1**), and it has overlapped conidia and apical appendage characteristics with *N. chrysea*, whereas *N. umbrinospora* produces umber-colored and comparatively wider conidia. Owing to the low nucleotide difference of ITS, *tub2*, and *tef1-α* sequences (below 1%) found among these strains, we synonymized *N. umbrinospora* under *N. chrysea.* Another new record of *Neopestalotiopsis* was described; the strain SDBR-CMU517 was phylogenetically located in the same clade with *N. cercidicola*, *N. terricola*, and *N. haikouensis* (**Figure 1**). The nucleotide comparison of ITS, *tub2*, and *tef1-α* sequence among those strains was insignificant (lower than 1%) (**Table 5**). Moreover, the strains of *N. terricola* showed high variation of conidial dimensions and color intensities on different host substrates (**Table 6**). We also observed this phenotypic variation of conidia in the new strain (**Figure 5G**). Therefore, we designated our strain as *N. haikouensis* and synonymized *N. terricola* and *N. cercidicola* under *N. haikouensis* based on phylogenetic and morphological congruency.

Moreover, a new isolate of *Pestalotiopsis* was reported; the strain SDBR-CMU518 has a close phylogenetic relationship with *P. jinchanghensis* and *P. zhaoqingensis* (**Figure 2**). The conidial morphology of this isolate is more similar to the ex-type of *P. jinchanghensis*, whose apical appendages are not branched, whereas *P. zhaoqingensis* has branched apical appendages and relatively shorter conidia. Based on their nucleotide differences being not significant (below 1.5%) coupled with phylogenetic data, we considered our new strain to belong to *P. jinchanghensis* and synonymized *P. zhaoqingensis* under *P. jinchanghensis*.

Another new species described in this study is *Pseudopestalotiopsis iteae*, which is characterized by the production of conidia with brown concolorous median cells, constricted at septa with spatulate apical appendage. The novel strains (SDBR-CMU514, SDBR-CMU519, SDBR-CMU520, SDBR-CMU521, SDBR-CMU522, SDBR-CMU523, and SDBR-CMU524) constitute a distinct clade and sister to *Ps. theae*. Furthermore, the nucleotide variation of *tef1-α* among the ex-type strains of *Ps. theae* and *Ps. iteae* was greater than 1.5% (**Table 7**), validating it as a new species based on the recommendation of Jeewon and Hyde (2016) and Chethana et al. (2021). Notably, some strains of *Ps. iteae* exhibited highly variable conidial shapes and sizes (**Figures 8I–K**), which were also observed in *Ps. theae* strains.

In summary, the three-locus phylogeny (ITS, *tub2*, and *tef1-α*) does not provide a strong support for species boundaries of pestalotioid fungi. Hence, the morphological characteristics and nucleotide polymorphisms were herein applied to elucidate their interspecific differences. The morphological characteristics of pestalotioid fungi are always similar and difficult to distinguish from each other. Likewise, their morphological characteristics showed a high intraspecific variation under different conditions of fungal growth media, hosts, temperatures, and other environmental factors (personal observation). Thus, it is unreliable to define new pestaloioid species using host, culture, and morphological comparisons. The significant phenotypic and genetic variations among pestalotioid species and strains have been addressed in recent studies (Norphanphoun et al., 2019; Li et al., 2022; Sun et al., 2023; Koodalugodaarachchi et al., 2024), concurring with the present study. Our phylogeny also contained unclear phylogenetic lineages including *Neopestalotiopsis chrysea*, *N. haikouensis*, *Pestalotiopsis jinchanghensis*, *Pseudopestalotiopsis theae*, and *Ps. iteae*, representing them as cryptic species. Therefore, further collections and more cultures are required for the reevaluation of intra- and interspecific relationships among these species. Moreover, additional informative loci, such as *rpb2*, and whole genome analysis are absolutely needed to evaluate the better taxonomic resolution of this fungal group.

The majority of pestalotioid taxa have been reported as plant pathogens (Maharachchikumbura et al., 2011; 2014; Peng et al., 2022; Giri and Pradhan, 2023; Hsu et al., 2024). Likewise, the species identified in this study—*Neopestalotiopsis chrysea*, *N. haikouensis*, and *Pestalotiopsis jinchanghensis*—were previously documented as causative agents of disease in significant plants (Liu et al., 2017; Shi et al., 2017; Jiang et al., 2021; Wu et al., 2021; Li et al., 2022; Zhang et al., 2022; 2024). Notably, some species exhibited distinct life modes including endophytes, saprobes, and phytopathogens. For example, *N. chrysea* was initially found as saprobes on unknown plants (Maharachchikumbura et al., 2012; 2014) and later reported as pathogens on blueberry and pecan in China (Shi et al., 2017; Wu et al., 2021). *Pestalotiopsis jinchanghensis* was introduced by Liu et al. (2017) as tea pathogen and was recently found as saprobes on unknown plants in China (Dong et al., 2023). Moreover, some species have a broad range of host association; for example, *N. haikouensis* could infect various plant taxa, such as *Castanea mollissima, Cercis chinensis, Ilex chinensis, Paeonia suffruticosa*, and *Olea europaea* (Jiang et al., 2021; Li et al., 2022; Zhang et al., 2022; 2024). These studies emphasize their abilities of lifestyle shift and host adaptation. Since we discovered endophytic pestalotioid fungi from *Itea*, further studies should be conducted to determine their pathogenicity as they may be a potential source of diseases.

To date, few fungi on the *Itea* species have been recorded (Farr et al., 2021), including only two pestalotioid species (*Pestalotiopsis acacia* on *Itea chinensis* var. *oblonga* from China and *P. gracilis* on *I. oldhamii* from Japan). Our study indicates that *Itea* contains a significant diversity of pestalotioid species. Moreover, we have observed that distinct pestalotioid species seem to prefer responsive tissues of *Itea*. Most strains of *Pseudopestalotiopsis* were found on leaves of *I. riparia*. Most *Pestalotiopsis* and *Neopestalotiopsis* species were found on stems of *I. japonica* and *I. riparia*. Only one species of *Pseudopestalotiopsis* and one *Neopestalotiopsis* species were found on roots of *I. riparia*. Our findings enhance the knowledge of taxonomic diversity of pestalotioid fungi in Thailand and highlight that *Itea* spp. could offer a high degree of undetermined fungal diversity. Since *Itea* is a significant source of rare sugar such as D-allulose, future studies are needed to examine the new endophytic fungal strains for the biosynthesis of rare sugar and other secondary metabolites. This would provide useful applications for these novel microbial resources in the pharmaceutical and food industries as well as in sustainable agriculture.

## Data Availability

The datasets presented in this study can be found in online repositories. The names of the repository/repositories and accession number(s) can be found below: https://www.ncbi.nlm.nih.gov/genbank/ ITS: PQ521226, PQ521227, PQ521228, PQ521229, PQ521230, PQ521231, PQ521232, PQ521233, PQ521234, PQ521235, PQ521236; tub2: PQ560699, PQ560700, PQ560701, PQ560702, PQ560703, PQ560704, PQ560705, PQ560706, PQ560707, PQ560708, PQ560709; *tef1-α*: PQ529177, PQ529178, PQ529179, PQ529180, PQ529181, PQ529182, PQ529183, PQ529184, PQ529185, PQ529186, PQ529187.
